# Fluorinated‐Squaramide Covalent Organic Frameworks for High‐Performance and Interference‐Free Extraction of Synthetic Cannabinoids

**DOI:** 10.1002/advs.202302925

**Published:** 2023-10-09

**Authors:** Yueru Shi, Ruolun Xu, Shaohan Wang, Juan Zheng, Fang Zhu, Qingkun Hu, Junlong Huang, Gangfeng Ouyang

**Affiliations:** ^1^ MOE Key Laboratory of Aquatic Product Safety/KLGHEI of Environment and Energy Chemistry School of Chemistry Sun Yat‐sen University Guangzhou 510275 China; ^2^ Anti‐Drug Technology Center of Guangdong Province Guangdong Provincial Key Laboratory of Psychoactive Substances Monitoring and Safety Guangzhou 510535 China; ^3^ SGS‐CSTC Standards Technical Services Co., Ltd. Guangzhou 510670 China

**Keywords:** covalent organic frameworks, extraction, synthetic cannabinoids

## Abstract

Synthetic cannabinoids (SCs), one of the largest groups of new psychoactive substances (NPSs), have emerged as a significant public health threat in different regions worldwide. Analyzing SCs in water samples is critical to estimate their consumption and control. However, due to their low background concentration and the coexistence of complex matrix, the selective and effective enrichment of SCs is still challenging. In this study, a series of fluorinated‐squaramide‐based covalent organic frameworks (COF: FSQ‐2, FSQ‐3, and FSQ‐4) are synthesized, and the as‐prepared FSQ‐4 exhibits strong affinity to different SCs. The proper pore size (1.4 nm) and pre‐located functional groups (hydrogen‐bond donors, hydrogen‐bond acceptors, and fluorophilic segments) work synergistically for efficient SCs capture. Remarkably, when coupled FSQ‐4 with solid‐phase microextraction (SPME), trace‐level (part per trillion, 10^−9^) determination of 13 SCs can be easily achieved, representing one of the best results among NPS analyses, and the excellent extraction performance can be maintained under various interfering conditions.

## Graphical abstract

Due to the rapid emergence of synthetic cannabinoids (SCs) and matrix interference, efficient extraction of different SCs is challenging. Herein, a covalent organic framework (FSQ‐4) is prepared from a trifluoromethyl‐decorated squaramide and 2,4,6‐triformylphloroglucinol, which possesses abundant hydrogen‐bond sites, fluorophilic segments, and high surface area. By coupling FSQ‐4 with solid‐phase microextraction, high‐performance and interference‐free extraction of 13 SCs is easily achieved.

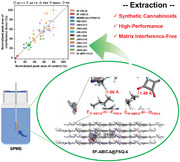

## 1 Introduction

Drug abuse is a long‐standing public health issue around the world. According to the United Nations Office on Drugs and Crime (UNODC), the number of people using illegal drugs has risen by 26% to 284 million over the past decade, and over one thousand kinds of new psychoactive substances (NPS) have been reported to the UNODC Early Warning Advisory by 137 countries and territories.^[^ ^1^, ^2^, ^3^, ^4^ ^]^ Among them, synthetic cannabinoids (SCs) are the most widely used, with an estimated 192 million users in 2018^[^ ^2^, ^5^ ^]^; they act on the same brain receptors as tetrahydrocannabinol (THC) but have more potent pharmacological effects than natural cannabinoids, leading to dangerous side effects such as kidney failure, heart attack, confusional psychoses and in some cases, death.^[^ ^6^, ^7^ ^]^ Many countries, including the United States,^[^ ^6^ ^]^ New Zealand_,_^[^ ^8^ ^]^ and China,^[^ ^9^ ^]^ have amended laws to ban these addictive chemicals, and there is an urgent demand for more sensitive and advanced detection technology to monitor trace‐level SCs.^[^ ^10^, ^11^, ^12^ ^]^

Analyzing SCs is challenging considering their low background level, and the complex matrix (such as proteins, organic acids, carbohydrates, and humic acid) in real‐world samples further hinders sensitive detection. At present, the detection of SCs has been dominated by tandem and high‐resolution mass spectrometry (MS) coupled with gas chromatography (GC) or liquid chromatography (LC),^[^ ^13^ ^]^ and different sample pre‐treatment techniques have been reported for SCs enrichment. However, they are either time‐ or labor‐consuming and only suitable for several kinds of SCs. For example, liquid–liquid extraction (LLE) requires a large number of organic solvents and is incapable of highly water‐soluble SCs.^[^ ^14^, ^15^ ^]^ Solid‐phase extraction (SPE) can enrich SCs with different properties, but it also requires many organic solvents and is even more time‐consuming.^[^ ^16^, ^17^, ^18^, ^19^ ^]^ Few studies apply molecularly imprinted polymer (MIP) and metal–organic framework (MOF) to avoid the complex extraction process, but their sensitivity is relatively low.^[^ ^20^, ^21^ ^]^ In short, these state‐of‐art solutions can hardly achieve broad‐spectrum, highly sensitive, and interference‐free extraction simultaneously, which is critical to fulfilling the desire for accurate SCs analysis. Novel porous materials are attractive candidates for SCs extraction because of their high specific surface area and abundant functional groups.^[^ ^22^, ^23^, ^24^, ^25^ ^]^ We envisioned that a rationally designed adsorbent and an advanced sample pre‐treatment technology could fulfill these demands.

As a family of organic crystalline porous materials with high surface areas, permanent porosity, and high stability, covalent‐organic frameworks (COFs) have become promising candidates in task‐specific separation.^[^ ^26^, ^27^, ^28^ ^]^ According to our previous work, a tailor‐made fluorinated‐squaramide can simultaneously introduce hydrogen bond receptors, donors, and hydrophobic segments as required.^[^ ^29^ ^]^ In this work, we synthesized three new fluorinated‐squaramide‐based COFs (FSQ‐2, FSQ‐3, FSQ‐4) by combining trifluoromethyl‐decorated squaramide with different aldehydes, and the as‐prepared FSQ‐4 showed excellent affinities to different SCs. When coupled with the solid‐phase microextraction (SPME), the custom FSQ‐4 SPME fiber can selectively and efficiently extract 13 SCs from real‐world samples, and trace‐level determination (<0.11 ng L^−1^) can be readily achieved. Theoretical simulation reveals that the proper pore size (≈1.4 nm) and trifluoromethyl‐decorated squaramide are key features that endow its superior performance.

## 2 Results and Discussion

### 2.1 Synthesis and Characterizations of Fluorinated‐Squaramide COFs (FSQ‐Xs)

**Scheme 1:**
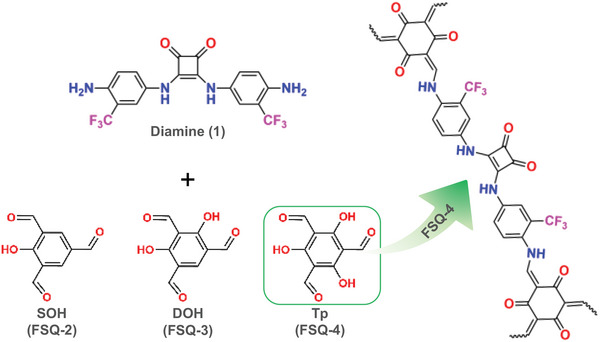
The schematic diagram for the synthesis of FSQ‐2, FSQ‐3, and FSQ‐4.

Based on 3,4‐bis[4‐amino‐3‐(trifluoromethyl)anilino]cyclobut‐3‐ene‐1,2‐dione (1) (diamine 1) and different aldehydes (2‐hydroxybenzene‐1,3,5‐tricarbaldehyde (SOH), 2,4‐dihydroxybenzene‐1,3,5‐tricarbaldehyde (DOH), and 2,4,6‐triformylphloroglucinol (*T*p)), three new fluorinated‐squaramide COFs (FSQ‐2, FSQ‐3, FSQ‐4) were synthesized by the [3+2] condensation (**Scheme** **1**). Details can be found in the Experimental Section. Three COFs with an irreversible keto‐enamine structure could be obtained under an acetic acid concentration of 6 m, reaction temperature of 120 °C, and reaction time of 3 days (Table S1, Supporting Information).

**Figure 1:**
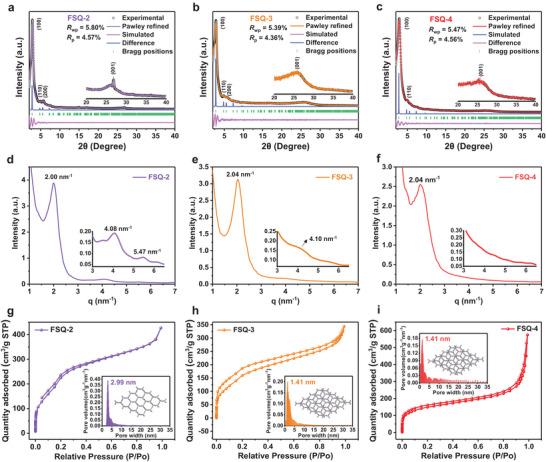
Experimental, Pawley‐refined, and simulated PXRD patterns of a) FSQ‐2 (AA stacking), b) FSQ‐3 (AB stacking), and c) FSQ‐4 (AB stacking). 1D SAXS profiles of d) FSQ‐2, e) FSQ‐3, and f) FSQ‐4. Nitrogen adsorption–desorption isotherms and pore volume histograms of g) FSQ‐2, h) FSQ‐3, and i) FSQ‐4.

Powder X‐ray diffraction (PXRD) pattern and simulation results indicated that FSQ‐2 exhibited an AA stacking model (**Figure** **1**a; Figure S1a and Table S2, Supporting Information). Diffraction peaks at *d*‐spacing of (2*θ*) 31.81 Å (2.84°), 18.57 Å (4.84°), and 15.90 Å (5.68°) were also observed, which were assigned to the (100), (110), and (200) diffraction planes of FSQ‐2, respectively (Figure 1a). Additional weak reflection at ≈26.82° (2*θ*) was assigned to the (001) facet; this confirmed the crystalline π–π stacked 2D structure of FSQ‐2 (Figure 1a). Moreover, the stacking patterns of the as‐prepared COFs varied with the number of hydroxyl groups in the aldehydes, FSQ‐3 and FSQ‐4 conformed to the AB stacking model. The PXRD pattern of FSQ‐3 showed diffraction peaks at *d*‐spacing of (2*θ*) 30.68 Å (2.80°), 18.30 Å (4.84°), and 15.34 Å (5.68°), corresponding to the (100), (110), and (200) diffraction planes, respectively (Figure 1b; Figure S1b and Table S3, Supporting Information). Similarly, the diffraction peaks of FSQ‐4 at *d*‐spacing (2*θ*) of 30.57 Å (2.84°) and 17.85 Å (4.87°) were ascribed to (100) and (110) diffraction planes, respectively (Figure 1c; Figure S1c and Table S4, Supporting Information). The broad peaks at 2*θ* angles of 26.32° and 25.27° belonged to the (001) planes of FSQ‐2 and FSQ‐3, respectively, demonstrating the π−π stacking of the COF layers. Rietveld refinement also verified the structural features of the as‐synthesized COFs (Figure S1d–f, Supporting Information).

To further verify the structure of the three COFs, small‐angle X‐ray scattering (SAXS) experiments were conducted. The SAXS profile of FSQ‐2 displayed scattering signals at *q* = 2.00, 4.08, and 5.47 nm^−1^, corresponding to *d*‐spacing of 3.14 nm (100), 1.54 nm (200), and 1.15 nm (210), respectively (Figure 1d). These results are consistent with the planes obtained from the PXRD data, further confirming the formation of the FSQ‐2 crystalline structure. SAXS peaks of FSQ‐3 at *q* = 2.04 and 4.10 nm^−1^ were assigned to *d*‐spacing of 3.08 nm (100) and 1.53 nm (200), respectively (Figure 1e). The SAXS profile of FSQ‐4 only displayed a scattering peak at *q* = 2.04 nm^−1^, corresponding to a *d*‐spacing of 3.08 nm (100) (Figure 1f). In short, the crystallinity of the COFs decreased slightly with an increase in keto‐enamine transformation.^[^ ^30^ ^]^

Different stacking models were also confirmed by the nitrogen adsorption–desorption isotherms (Figure 1g–i). The Brunauer–Emmett–Teller (BET) surface areas of FSQ‐2, FSQ‐3, and FSQ‐4 were calculated as 834, 596, and 534 m^2^ g^−1^, respectively. Notably, the pore size distribution of said COFs, as determined by density functional theory (DFT), shows that the mode pore transformed from mesoporous (FSQ‐2:2.99 nm) to microporous (FSQ‐3 and FSQ‐4:1.41 nm), which is consistent with the stacking models of the three COFs (Figure S1, Supporting Information). Moreover, the steep front in the nitrogen adsorption–desorption isotherms of FSQ‐4 indicated the presence of some mesopores in this material (Figure 1i); FSQ‐4 also showed the highest cumulative pore volume among the three COFs (FSQ‐4:0.604, FSQ‐3:0.451, and FSQ‐2:0.591 cm^3^ g^−1^) (Figure S2, Supporting Information).

**Figure 2:**
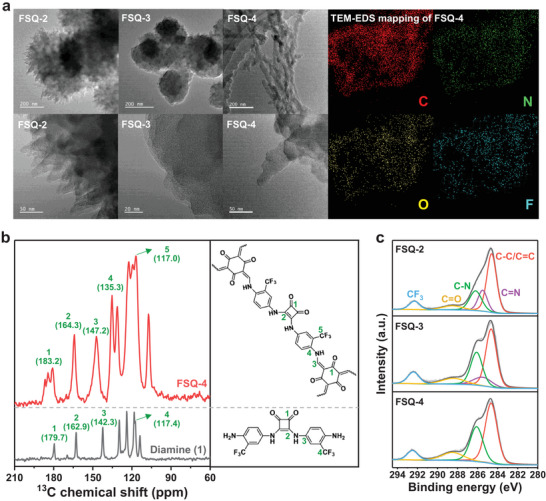
a) TEM images of FSQ‐2, FSQ‐3, and FSQ‐4 from spherical aberration corrected TEM and the corresponding elemental TEM‐EDS mappings of FSQ‐4. b) ^13^C solid‐state NMR spectra of diamine (1) and FSQ‐4. c) C 1s XPS spectra of FSQ‐2, FSQ‐3, and FSQ‐4.

Scanning electron microscopy (SEM) and transmission electron microscopy (TEM) images revealed the sphere‐like morphologies of FSQ‐2 and FSQ‐3, whereas FSQ‐4 exhibited a fiber‐like shape (Figure S3a–f, Supporting Information; **Figure** **2**a). High‐resolution TEM (HRTEM) images indicated that the three COFs have lamellate structures, which might be due to the π−π stacking of COF layers (Figure 2a; Figure S3g–i, Supporting Information). The presence of different and uniformly distributed elements in the three types of COFs was verified by energy‐dispersive spectroscopy (EDS) mapping (Figure 2a; Figure S3j,k, Supporting Information).

Because various functional groups were integrated into the framework, solid‐state ^13^C cross‐polarization magic‐angle spinning (CP/MAS) nuclear magnetic resonance (NMR) was applied to validate the functional groups in the FSQ‐Xs skeleton. For FSQ‐4, a broad peak (≈183.2 ppm) confirmed the presence of the β‐ketoenamine carbonyl carbon (C═O) (Figure 2b), and signals at 147.2 and 117.0 ppm were assigned to the amine (C─NH) and trifluoromethyl (CF_3_) groups, respectively. The signals (≈181 ppm) of FSQ‐2 and FSQ‐3 also confirmed the presence of the β‐ketoenamine carbonyl carbon (C═O) (Figure S4, Supporting Information). In addition, representative peaks at 292.4, 288.5, and 286.2 eV in the X‐ray photoelectron spectroscopy (XPS) spectra further confirmed the presence of trifluoromethyl, carbonyl, and amine groups in these materials, respectively (Figure 2c). The peak areas of C─N and C═O in FSQ‐2, FSQ‐3, and FSQ‐4 gradually increased, indicating different degrees of enol‐to‐keto tautomerization (Table S5, Supporting Information), which also influenced their water contact angles (Figure S2l–n, Supporting Information). As shown in the Fourier transform infrared (FT‐IR) spectra, a shoulder peak of the FSQ‐Xs located at 1642 cm^−1^ overlapped with a strong peak at 1578 cm^−1^, which arose from C═O and C═C stretching, respectively (Figure S5a–c, Supporting Information). Additionally, good thermal stability (up to 280 °C) of the FSQ‐4 was verified by thermogravimetric analysis (TGA), whereas FSQ‐2 and FSQ‐3 began to decompose at 220 °C (Figure S6, Supporting Information).

### 2.2 Extraction of SCs by FSQ‐4‐Based SPME Coating

SPME is a green pre‐treatment technology that integrates separation, enrichment, and purification into a single step.^[^ ^31^, ^32^ ^]^ As a solvent‐free and non‐exhaustive extraction technique, SPME minimizes both adsorbent and organic solvent consumption, and its performance is dictated by the coating material.

**Figure 3:**
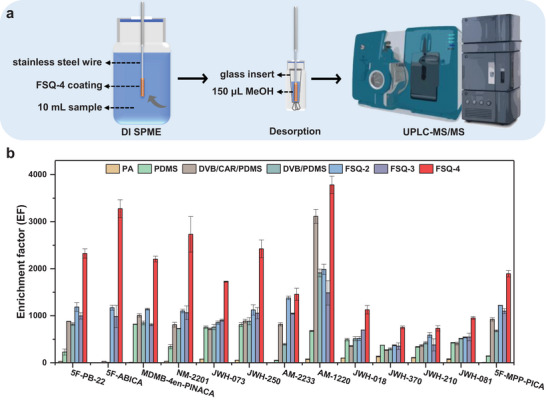
a) Schematic diagram of the extraction procedure. b) Comparison results of enrichment factors toward SCs using commercial PA, PDMS, DVB/CAR/PDMS, DVB/PDMS, FSQ‐2, FSQ‐3, and FSQ‐4 fibers (10 mL, 5 µg L^−1^).

The as‐prepared COFs were first fabricated as SPME coatings (details in the Experimental Section and Figure S7, Supporting Information), and their extraction efficiencies toward 13 SCs were evaluated in deionized water by direct immersion (DI) SPME with ultra‐performance liquid chromatography‐tandem mass spectrometry (UPLC‐MS/MS) (**Figure** **3**a). Commercial SPME fibers, including polydimethylsiloxane/divinylbenzene (PDMS/DVB), polyacrylate (PA), PDMS, and DVB/carboxen/PDMS (DVB/CAR/PDMS), were selected for comparison.

As can be seen from the results, the enrichment factors (EFs) of the FSQ‐4 fiber toward the 13 SCs ranged from 732 to 3784 (Figure 3b), which was 1.7 to 2245.5 times better than those of commercial fibers, highlighting its superior performance in trace‐level SCs enrichment. Moreover, the extraction performance of the FSQ‐4 fiber was also 1.1 to 3.3 times higher than those of FSQ‐2 and FSQ‐3, respectively. Given the different molecular sizes and distinctive log*P* values (varying across four orders of magnitude) of the aforementioned SCs, these results demonstrated that the combination of adequate pores and functional groups is critical for effective SCs capture (Figure 1i; Table S6, Supporting Information).

### 2.3 Matrix Interfering Resistance of the FSQ‐4 Fiber

Selective (or matrix‐free) extraction is crucial in real‐world applications because the concentrations of various interfering substances are usually much higher than those in SCs. In most cases, competitive adsorption inhibits target capture, and complex pretreatments (such as acidification) are necessary. SPME is an attractive technique because it integrates sample purification and target capture in a single step.

**Figure 4:**
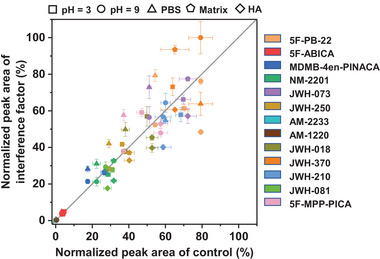
Extraction efficiencies of FSQ‐4 fiber toward SCs under pH interference, ion interference, and interfering substances.

To study anti‐interference performance, the FSQ‐4 fiber was used to extract SCs from various solutions, including solutions with different pH values (3–9), phosphate‐buffered saline (PBS) (10 mmol L^−1^), humic acid (20 mg L^−1^), and a mixture of common interfering substances (amino acids, proteins, organic acids, and carbohydrates). The extraction efficiencies of these complex matrices agreed well with those of deionized water, confirming its good selectivity and anti‐interference ability (**Figure** **4**). These results are attributed to the strong affinity of FSQ‐4 for SCs and the size‐exclusion effect of the micropores. Thus, the developed FSQ‐4 fiber could directly and selectively extract SCs from untreated water samples.

### 2.4 Method Validation and Real Sample Determination

The parameters affecting the SPME efficiency of the custom FSQ‐4 fiber, including extraction time, desorption time, and desorption solvent, were optimized. After optimization, an extraction time of 40 min, a desorption time of 15 min, and MeOH as a desorption solvent were chosen as the best combination (Figure S8, Supporting Information); accordingly, a good analytical performance was obtained (**Table** **1**). The high enrichment factors (EFs) of the FSQ‐4 fiber for all target SCs resulted in low limits of detection (LODs). The LODs were all below 0.11 ng L^−1^, which is much lower than most reported LODs (Table S7, Supporting Information); the performances of the FSQ‐4 fiber remained at high levels after multi‐cycle extraction (Figure S9, Supporting Information). In addition, the wide linear range and satisfactory reproducibility (single fiber and fiber‐to‐fiber) guaranteed the practicality of the developed method (Table 1), indicating that it has great potential for ultra‐trace analysis of SCs in real wastewater samples. Urban wastewater from sewage treatment plants (STPs) is an economical and accessible source of real‐time epidemiological data, so the developed method was used to evaluate wastewater recovery in three cities in southern China. The recoveries of 13 SCs ranged from 80.5% to 113.9%, indicating the reliability of the developed method (Table S8, Supporting Information).

**Table 1:** The method evaluation results of SCs by FSQ‐4 fiber coupling to UPLC‐MS/MS.

Analytes	Linear range [ng L^−1^]	R^2^	EF	LODs [ng L^−1^]	RSD [%]	
					Single fiber, *n* = 6	Fiber‐to‐fiber, *n* = 3
5F‐PB‐22	1–10 000	0.9975	2324	0.067	6.0	1.8
5F‐ABICA	1–10 000	0.9942	3277	0.068	4.7	5.6
MDMB‐4en‐PINACA	1–10 000	0.9994	2204	0.075	4.1	3.3
NM‐2201	1–10 000	0.9916	2730	0.063	11	7.7
JWH‐073	1–10 000	0.9935	1725	0.021	2.3	4.1
JWH‐250	1–10 000	0.9976	2422	0.012	14	6.5
AM‐2233	10–50 000	0.9958	1456	0.089	8.5	9.6
AM‐1220	10–50 000	0.9989	3784	0.11	12	7.2
JWH‐018	1–10 000	0.9927	1127	0.018	5.4	8.8
JWH‐370	1–10 000	0.9967	752	0.014	16	6.5
JWH‐210	1–10 000	0.9932	732	0.024	7.8	2.4
JWH‐081	1–10 000	0.9970	953	0.028	12	6.9
5F‐MPP‐PICA	1–10 000	0.9935	1895	0.017	6.1	2.2

### 2.5 Mechanism Study

TEM‐EDS mapping and XPS were performed to determine the underlying adsorption mechanism. Taking AM‐2233 as an example, a uniform distribution of iodine (the characteristic element in AM‐2233) in FSQ‐4 was observed after adsorption (Figure S10a, Supporting Information). New peaks representing C–I at 620.7 eV (I 3d_5/2_) and 632.3 eV (I 3d_3/2_) also appeared in the XPS spectra, which confirmed the adsorption of AM‐2233 by FSQ‐4 (Figure S10b,c). Moreover, the adsorption isotherms (Langmuir and Freundlich models) of FSQ‐4 toward the 13 SCs were plotted (Figures S11 and S12, and Table S9, Supporting Information). The Langmuir model described the experimental data well, exhibiting high correlation efficiencies; this indicated that the adsorption of the SCs mainly occurred via monolayer adsorption. The maximum adsorption capacity *(Q*_max_) of 13 the SCs ranged from 171.3 to 289.2 mg g^−1^, and their Langmuir adsorption constant (*K*_L_) was above 806.07 L mmol^−1^, demonstrating the excellent adsorption capacity of FSQ‐4.

Theoretical simulations were performed to probe the interactions between the SCs and FSQ‐4. The molecular sizes of the 13 SCs ranged from 1.31 to 1.69 nm, which is close to the pore size of FSQ‐4 (Figure S13, Supporting Information). Subsequently, the surface electrostatic potentials of the 13 SCs were calculated. SCs are a class of compounds with an apparent electron distribution; their aromatic rings and halogen atoms are relatively electron‐rich, whereas their alkane chains are electron‐deficient (Figure S14 and Table S6, Supporting Information). They also contain hydrophobic (aromatic rings, alkane chains, and indolyl groups) and hydrophilic (amide and carbonyl groups) segments. These structural and electrostatic features are important indicators for adsorbent design applications.

The electrostatic properties of FSQ‐4 were also calculated. Numerous trifluoromethyl (─CF_3_) and carbonyl (C═O) groups were introduced into the framework using trifluoromethyl‐decorated squaramide. Owing to the strong electronegativity of fluorine and oxygen, the local electron density of the FSQ‐4 channel increased significantly (Figure S15a, Supporting Information). Furthermore, as there was abundant hydroxyl (─OH) groups on the aldehyde precursor (Tp), more electron‐rich groups (C═O) appeared in the framework after the keto‐enamine conversion (Figure S15a, Supporting Information). As a result, the local electron density of FSQ‐4 was higher than those of FSQ‐2 and FSQ‐3 (Figure S15b,c, Supporting Information). More precisely, the atomic charge and dipole moment were calculated to reflect the electron distribution across the framework. Due to the strong electron‐withdrawing properties of oxygen (−0.277–−0.281e) and fluorine (−0.094–−0.112e), squaramide and trifluoromethyl groups in these three COFs exhibited significant dipole moments of 8.92–9.20 and 0.80–0.90 D, respectively (Table S10 and Figure S15d–f, Supporting Information). The dipole moment of the amine (C─NH) was smaller than that of the imine (C═N) (Table S10 and Figure S15d–f, Supporting Information). Moreover, the electron density of the β‐ketoenamine carbonyl oxygen (−0.292e) in FSQ‐4 was significantly higher than those of the hydrogen (0.055–0.058e) in the corresponding positions and the imine nitrogen (−0.156–−0.170e) in FSQ‐2 and FSQ‐3. These results indicated that enol‐to‐keto tautomerism increased the local electron density of the COF skeleton. Therefore, FSQ‐4 can provide diverse binding sites to recognize various SCs via non‐covalent interactions, such as hydrogen bonding.

**Figure 5:**
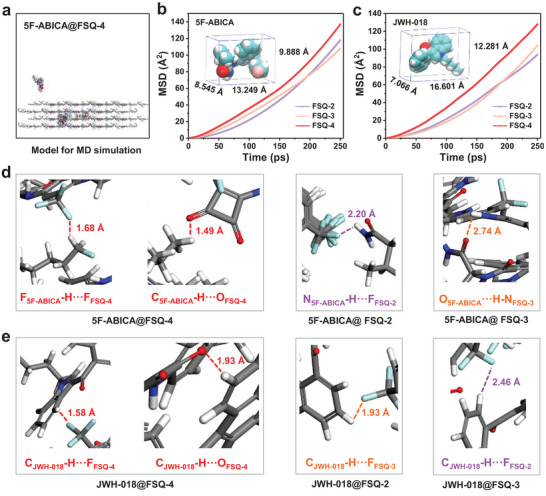
a) Model of 5F‐ABICA@FSQ‐4 for MD simulation. MSD plots of b) 5F‐ABICA and c) JWH‐018 in FSQ‐2, FSQ‐3, and FSQ‐4, respectively. Molecular dimensions (insert) of b) 5F‐ABICA and c) JWH‐018. d,e) Representative snapshots containing the distribution.

In order to further illustrate the extraction mechanism, molecular dynamics (MD) simulations were carried out. In this regard, two types of SCs with (5F‐ABICA) and without (JWH‐018) fluorine were selected as representatives, and their adsorption behaviors were simulated in water (**Figure** **5**a; Figure S16, Supporting Information). The mean square displacements (MSD) of 5F‐ABICA and JWH‐018 in the three COFs increased over time, indicating that they moved from the aqueous solution to the material (Figure 5b,c). Notably, the trajectories exhibited the fastest growth in FSQ‐4 (Figure 5b,c), and the derived diffusion coefficients (D) were significantly higher than those of FSQ‐2 and FSQ‐3 (Figure S17, Supporting Information). These results indicate that the adsorption of the two targeted SCs was kinetically favored by FSQ‐4.

Conformational changes during MD simulations were also studied. 5F‐ABICA and JWH‐018 tended to be located near the trifluoromethyl (─CF_3_) and carbonyl (C═ O) groups (Figure 5d,e). The close distances indicate strong non‐covalent interactions, such as hydrogen bonds and hydrophobic interactions. The higher local electron density in the FSQ‐4 skeleton promoted the kinematic activity of the analytes and enhanced aperture utilization, further confirming the critical roles of the carbonyl groups and micropores in the adsorption process. Overall, the abundant trifluoromethyl groups (─CF_3_), carbonyl groups (C═O), and matching micropores on FSQ‐4 played a vital role in the SCs extraction process.

## 3 Conclusion

In summary, by virtue of different functional groups from the rationally designed linkers and the micro‐level pores from the interlayer stacking, the as‐prepared FSQ‐4 was an efficient extraction material for different SCs, in which the hydrogen bond and hydrophobic effect play pivot roles. Moreover, a highly sensitive and anti‐inferring sample pre‐treatment method was established by coupling FSQ‐4 and SPME, beyond which trace‐level (part per trillion, 10^−9^) detection of 13 SCs can be readily achieved. These results highlighted the promise of COFs in task‐specific separation, especially in simplifying traditional analytical processes that are critical for drug enforcement and public health departments.

## 4 Experimental Section

### Synthesis of FSQ‐2

The 3,4‐bis[4‐amino‐3‐(trifluoromethyl)anilino]cyclobut‐3‐ene‐1,2‐dione (1) (diamine (1)) was synthesized according to the previous works.^[^ ^29^ ^]^ Diamine (1) (25.8 mg, 0.06 mmol) and SOH (7.13 mg, 0.04 mmol) were added to a 10 mL Pyrex tube. Then 1,2‐dichlorobenzene (*o*‐DCB, 1 mL) and N, N‐dimethyl acetamide (DMAc, 10 drops) were added to the Pyrex tube, and the mixture was ultrasonic until even. The catalyst ratio, reaction temperature, and reaction time were screened according to Table S1 (Supporting Information). After the reaction, the product was filtered and separated, then activated by Soxhlet extraction with tetrahydrofuran (THF). Finally, the yellow powder FSQ‐2 was obtained by vacuum drying.

### Synthesis of FSQ‐3 and FSQ‐4

Similarly, the SOH content in the synthesis steps of FSQ‐2 was changed to DOH (7.77 mg, 0.04 mmol) and Tp (8.41 mg, 0.04 mmol), respectively.

### Preparation of FSQ‐Xs SPME Fiber

The stainless steel wires (470 µm) were cut into ≈3.5 cm segments and ultrasonically cleaned by acetone, ethanol, and ultrapure water in sequence for 10 min. Approximately 1.0 g PDMS was dispersed in a sample tube using 2.0 mL cyclohexane and ultrasonically dissolved to acquire a viscous solution. A pretreated SS wire was dipped into the viscous solution to coat a thin layer of PDMS. Then, the PDMS‐coated wire was rotated in COF powder to obtain a thin coating layer. The fiber coating was solidified at 80 °C for 30 min. All fibers were vibrated in methanol (20 mL) for 30 min before use.

### Method Development and Evaluation

The samples were prepared by dissolving certain SCs in 10 mL of deionized water or real sewage samples. Real samples of domestic sewage were collected from three different cities in southern China and provided by the National Anti‐Drug Laboratory Guangdong Regional Center. The vibration rate was set at 400 r min^−1^. The desorption solvents were MeOH, ACN, or EtOH, with a volume of 150 µL. The eluent was stored at −20 °C until analysis. The quantitation of targeted SCs was performed by a Waters Acquity UPLC system (Waters, USA) coupled with AB Sciex Triple Quad 5500+ triple‐quadrupole tandem mass spectrometer (ESI source, Applied Biosystems/MDS, USA). Waters Acquity UPLC BEN C18 column (2.1 mm × 100 mm, 1.7 µm, Waters, USA) was used for chromatographic separation. The mobile phases were 0.1% formic acid in water (solvent A) and ACN (solvent B). The optimized gradient and the monitoring transition of SCs are listed in Table S11 (Supporting Information). The chromatographic retention time of the SCs is listed in Table S12 (Supporting Information). The detection was conducted in the positive ion mode. The flow rate was 0.4 mL min^−1,^ and the injection volume was ten µL. Multiple reaction monitoring parameters are listed in Table S13 (Supporting Information). The *EF* was obtained by the following Equation:

(1) $$EF\;=\frac{C_{\mathrm{fiber}}}{C_{0}}\;$$

Where *C_0_* is the concentration of the sample, and *C*_fiber_ is the concentration of the fiber after extraction.

## Conflict of Interest

The authors declare no conflict of interest.

## Supporting information

Supporting Information

## Data Availability

The data that support the findings of this study are available in the supplementary material of this article.

## References

[1] United Nations Office on Drugs and Crime, World Drug Report 2022.

[2] A. O'Dowd , BMJ [Br. Med. J.] 2020, 369, m2631. 32605941 10.1136/bmj.m2631

[3] A. Peacock , R. Bruno , N. Gisev , L. Degenhardt , W. Hall , R. Sedefov , J. White , K. V. Thomas , M. Farrell , P. Griffiths , Lancet 2019, 394, 1668. 31668410 10.1016/S0140-6736(19)32231-7

[4] Current NPS threats, Vol. V, Oct. 2022, https://www.unodc.org/unodc/en/scientists/current-nps-threats.html.

[5] X. Fan , J. Zhang , X. Fu , B. Zhou , Z. Xu , H. Huang , S. Han , X. Li , Sci. Total Environ. 2022, 827, 154267. 35247413 10.1016/j.scitotenv.2022.154267

[6] E. Underwood , Science 2015, 347, 473. 25635070 10.1126/science.347.6221.473

[7] P. Pacher1 , S. Steffens , G. Haskó , T. H. Schindler , G. Kunos , Nat. Rev. Cardiol. 2018, 15, 151. 28905873 10.1038/nrcardio.2017.130

[8] K. Brown , BMJ 2011, 343, d5395. 21862549 10.1136/bmj.d5395

[9] C. Liu , Z. Hua , W. Jia , T. Li , Drug Test. Anal. 2022, 14, 307. 34694738 10.1002/dta.3185

[10] E. Zuccato , C. Chiabrando , S. Castiglioni , R. Bagnati , R. Fanelli , Environ. Health Perspect. 2008, 116, 1027. 18709161 10.1289/ehp.11022 PMC2516581

[11] L. Gent , R. Paul , Sci. Total Environ. 2021, 776, 146028.

[12] F. Asicioglu , M. K. Genc , T. T. Bulbul , M. Yayla , S. Z. Simsek , C. Adioren , S. Mercan , Water Res. 2021, 190, 116729. 33341037 10.1016/j.watres.2020.116729

[13] R. Roque‐Bravo , R. S. Silva , R. F. Malheiro , H. Carmo , F. Carvalho , D. D. Silva , J. P. Silva , Annu. Rev. Pharmacol. Toxicol. 2023, 63, 187. 35914767 10.1146/annurev-pharmtox-031122-113758

[14] A. J. Pandopulos , R. Bade , J. W. O'Brien , B. J. Tscharke , J. F. Mueller , K. Thomas , J. M. White , C. Gerber , Talanta 2020, 217, 121034. 32498913 10.1016/j.talanta.2020.121034

[15] A. J. Pandopulos , B. S. Simpson , R. Bade , J. W. O'Brien , M. K. Yadav , J. M. White , C. Gerber , Environ. Sci. Pollut. Res. 2021, 28, 59652. 10.1007/s11356-021-14921-3 34143389

[16] R. Bade , B. J. Tscharke , J. M. White , S. Grant , J. F. Mueller , J. O'Brien , K. V. Thomas , C. Gerber , Sci. Total Environ. 2019, 650, 2181. 30290358 10.1016/j.scitotenv.2018.09.348

[17] C. E. O'Rourke , B. Subedi , Environ. Sci. Technol. 2020, 54, 6661. 32356976 10.1021/acs.est.0c00250 PMC8014967

[18] T. Gao , P. Du , Z. Xu , X. Li , Sci. Total Environ. 2017, 575, 963. 27678045 10.1016/j.scitotenv.2016.09.152

[19] N. Salgueiro‐Gonzalez , S. Castiglioni , E. Gracia‐Lor , L. Bijlsma , A. Celma , R. Bagnati , F. Hernandez , E. Zuccato , Sci. Total Environ. 2019, 689, 679. 31279214 10.1016/j.scitotenv.2019.06.336

[20] J. Sánchez‐González , S. Odoardi , A. M. Bermejo , P. Bermejo‐Barrera , F. S. Romolo , A. Moreda‐Piñeiro , S. Strano‐Rossi , J. Chromatogr. A. 2018, 1550, 8. 29605179 10.1016/j.chroma.2018.03.049

[21] H. Martínez‐Pérez‐Cejuela , M. Conejero , P. Amorós , J. E. Haskouri , E. F. Simó‐Alfonso , J. M. Herrero‐Martínez , S. Armenta , Anal. Chim. Acta 2023, 1246, 340887. 36764780 10.1016/j.aca.2023.340887

[22] Y. Su , K. Otake , J. Zheng , S. Horike , S. Kitagawa , C. Gu , Nature 2022, 611, 289. 36352136 10.1038/s41586-022-05310-y

[23] C. Wang , Y. Wang , K. O. Kirlikovali , K. Ma , Y. Zhou , P. Li , O. K. Farha , Adv. Mater. 2022, 34, 2202287. 10.1002/adma.202202287 35790037

[24] Y. Su , Z. Wang , A. Legrand , T. Aoyama , N. Ma , W. Wang , K. Otake , K. Urayama , S. Horike , S. Kitagawa , S. Furukawa , C. Gu , J. Am. Chem. Soc. 2022, 144, 6861. 35315656 10.1021/jacs.2c01090

[25] Y. Su , B. Li , H. Xu , C. Lu , S. Wang , B. Chen , Z. Wang , W. Wang , K. Otake , S. Kitagawa , L. Huang , C. Gu , J. Am. Chem. Soc. 2022, 144, 18218. 36069433 10.1021/jacs.2c05701

[26] X. Xiong , Z. Yu , L. Gong , Y. Tao , Z. Gao , L. Wang , W. Yin , L. Yang , F. Luo , Adv. Sci. 2019, 6, 1900547. 10.1002/advs.201900547 PMC6702651 31453066

[27] S. Zhang , Q. Yang , C. Wang , X. Luo , J. Kim , Z. Wang , Y. Yamauchi , Adv. Sci. 2018, 5, 1801116. 10.1002/advs.201801116 PMC6299720 30581707

[28] Z. Chen , X. Li , C. Yang , K. Cheng , T. Tan , Y. Lv , Y. Liu , Adv. Sci. 2021, 8, 2101883. 10.1002/advs.202101883 PMC8529453 34411465

[29] J. Huang , Y. Shi , G. Huang , S. Huang , J. Zheng , J. Xu , F. Zhu , G. Ouyang , Angew. Chem., Int. Ed. 2022, 61, e202206749. 10.1002/anie.202206749 35599428

[30] M. Traxler , S. Gisbertz , P. Pachfule , J. Schmidt , J. Roeser , S. Reischauer , J. Rabeah , B. Pieber , A. Thomas , Angew. Chem., Int. Ed. 2022, 61, e202117738. 10.1002/anie.202117738 PMC9400916 35188714

[31] G. Ouyang , D. Vuckovic , J. Pawliszyn , Chem. Rev. 2011, 111, 2784. 21271696 10.1021/cr100203t

[32] S. Huang , J. Zheng , Q. Yang , G. Chen , J. Xu , Y. Shen , Y. Zhang , G. Ouyang , Adv. Sci. 2018, 5, 1800774. 10.1002/advs.201800774 PMC6299822 30581699

