# Supplementary material for: Fluorinated‐Squaramide Covalent Organic Frameworks for High‐Performance and Interference‐Free Extraction of Synthetic Cannabinoids
Source: Adv Sci (Weinh). 2023 Oct 9;10(32):2302925. doi: 10.1002/advs.202302925 (PMC10646270; doi:10.1002/advs.202302925)
Supplement: Supplementary file 1 — Supporting Information [file ADVS-10-2302925-s001.pdf]

ADVANCED SCIENCE
Open Access

Supporting Information

for *Adv. Sci.*, DOI 10.1002/advs.202302925

Fluorinated-Squaramide Covalent Organic Frameworks for High-Performance and Interference-Free Extraction of Synthetic Cannabinoids

Yueru Shi, Ruolun Xu, Shaohan Wang, Juan Zheng, Fang Zhu, Qingkun Hu, Junlong Huang* and Gangfeng Ouyang**

Supporting Information

Fluorinated-Squaramide Covalent Organic Frameworks for High-Performance and Interference-Free Extraction of Synthetic Cannabinoids

Yueru Shi, Ruolun Xu, Shaohan Wang, Juan Zheng, Fang Zhu, Qingkun Hu, Junlong Huang*, and Gangfeng Ouyang**

Y. Shi, S. Wang, J. Zheng, F. Zhu, G. Ouyang.
MOE Key Laboratory of Aquatic Product Safety/KLGHEI of Environment and Energy Chemistry, School of Chemistry, Sun Yat-sen University, Guangzhou, 510275, China.
E-mail: cesoygf@mail.sysu.edu.cn

R. Xu, Q. Hu.
Substances Monitoring and Safety, Guangdong Provincial Key Laboratory of Psychoactive, National Anti-Drug Laboratory Guangdong Regional Center, Guangzhou, 510535, China.

J. Huang.
SGS-CSTC Standards Technical Services Co., Ltd. Guangzhou, 510670, China.

Keywords: Covalent organic frameworks, synthetic cannabinoids, extraction

1. Chemicals and reagents

All chemicals and reagents are commercially available and used without further purification. 2-Hydroxybenzene-1,3,5-tricarbaldehyde (SOH), 2,4-dihydroxybenzene-1,3,5-tricarbaldehyde (DOH) and 2,4,6-triformylphloroglucinol (Tp) were bought from Jilin Chinese Academy of Sciences - Yanshen Technology Co., Ltd (Jilin, China). Zinc trifluoromethanesulfonate ($Zn(OTf)_2$), 1,2-dichlorobenzene (*o*-DCB), and N,N-dimethyl acetamide (DMAc) were purchased from Alfa Aesar Chemical Co., Ltd (Shanghai, China). Acetonitrile (ACN), ethanol (EtOH), tetrahydrofuran (THF), methanol (MeOH), and 2-(trifluoromethyl)-1,4-phenylenediamine were bought from Macklin biochemical technology Co., Ltd (Guangdong, China). Cyclohexane, acetone, hydrochloric acid, sodium hydroxide, methylbenzene, sodium phosphate dibasic dodecahydrate ($Na_2HPO_4 \cdot 12H_2O$), potassium chloride (KCl) and Potassium phosphate monobasic (KH_2PO_4) were purchased from Guangzhou Chemical Reagents Company (Guangdong, China). NaCl, $Na_2HPO_4 \cdot 12H_2O$, KCl, and KH_2PO_4 were used to prepare a phosphate buffer saline (PBS) solution. Palmitic acid, lauric acid, D-(+)-glucose 3,4-diethoxy-3-cyclobutane-1,2-dione, formic acid, glacial acetic acid (HAc) and N-methylpyrrolidone (NMP) were bought from Aladdin Chemistry Co., Ltd (Shanghai, China). Bovine albumin was bought from J&K Scientific Co., Ltd (Beijing, China). Amino acid standards (L-alanine, L-arginine, L-cystine, L-glutamic acid, L-histidine) were purchased from Sigma-Aldrich Co., Ltd (St. Louis, USA). Polydimethylsiloxane (PDMS) was prepared using the neutral silicone sealant from LESSO (Guangdong, China). Commercial PDMS (100 μm), Polyacrylate (PA, 85 μm), PDMS/Divinylbenzene (PDMS/DVB, 65 μm), and DVB/Carboxen/PDMS (DVB/CAR/PDMS, 50/30 μm) fibers were purchased from Supelco (Bellefonte, PA, United States). Stainless steel (SS) wires were provided by Component Supply Company (Fort Meade, MD, USA). Synthetic cannabinoids (NM-2201, 5F-ABICA, 5F-PB-22, MDMB-4en-PINACA, JWH-073, JWH-250, AM-2233, AM-1220, JWH-18, JWH 370, JWH-210, JWH-081, 5F-MPP-PICA) were supplied by National Anti-Drug Laboratory Guangdong Regional Center (Guangzhou, China).

2. Instruments

Quanta 200 scanning electron microscopy (SEM, USA) and JEOL2010 transmission electron microscope (TEM, Japan) were used to observe the morphology. Thermogravimetric analysis (TGA) was acquired from a TG 209 F3 Tarsus thermogravimetry (Netzsch, Germany). Water contact angle pictures were acquired from a DSA100 drop shape analyzer (Kruss, Germany). The X-ray photoelectron spectroscopy (XPS) spectra were obtained using a Thermo-VG

Scientific spectrometer (ESCALAB 250, USA). Crystallographic details were revealed using an Empyrean powder X-ray diffractometer (PXRD, Netherlands). Small-angle X-ray scattering (SAXS) measurements were carried out using Anton paar Saxsess MC2. An automated gas adsorption analyzer Autosorb-IQ3 (Quantachrome Instruments, America), was employed to measure the nitrogen adsorption-desorption isotherms at 77 K. The surface areas and pore size distributions were respectively obtained based on Brunauer Emmett Teller (BET) method and the density functional theory (NLDFT) method. The ^{13}C solid-state NMR spectra were recorded on a Bruker AVANCE NEO 600 spectrometer. Fourier transform infrared (FT-IR) spectra were recorded by Frontier Optica (PerkinElmer).

3. Isotherm experiments

A Langmuir adsorption isotherm was generated by plotting $1/Q_e$ versus $1/C$ in the following equation.

$$\frac{1}{Q_e} = \frac{1}{Q_{max}} + \frac{1}{Q_{max} K_L C}$$

where Q_e (mg/g) is the amount of pollutant adsorbed at equilibrium. Q_{max} (mg/g) is the maximum adsorption capacity of adsorbent at equilibrium. C (mmol/L) is the equilibrium concentration in the residual solution, and K_L (L/mmol) is the Langmuir adsorption constant.

The Freundlich adsorption isotherm is expressed as

$$Q_e = K_F C^{\frac{1}{n}}$$

Which can be represented in linear form as

$$\ln Q_e = \ln K_F + \frac{1}{n} \ln C$$

Where, Q_e (mg/g) is the amount of pollutant adsorbed at equilibrium. C (mg/L) is the equilibrium concentration in the residual solution. K_F (mg/g)(L/mg)$^{1/n}$ is the Freundlich isotherm constants. 1/n is the adsorption intensity.

4. Calculation method

Molecular dynamics: This part studied the diffusion performance of two molecules in COFs by molecular dynamics. A water box had four layers of COF. The dynamics part was calculated by the force plus module in Materials Studio software, where the force field was UFF[1]. The charge was distributed by the QEQ method. The electrostatic summation method was group based, and the van der Waals interaction energy summation method was atom based. The interactions were calculated by an atom-based method with a cutoff distance of 15.5 Å. The dynamics ensemble was NVT. The temperature was 300 K by a Nose-Hoover[2] thermostat.

The time step was 1 fs. The total simulation time was 1 ns, and a frame was output every 1000 steps. Finally, the whole trajectory file was processed to analyze the molecular diffusion performance.

Electrostatic potential: The Electrostatic potential of COF systems was studied using the $DMol^3$ module in Materials Studio. The electron exchange–function was calculated using Perdew-Burke–Ernzerhof (PBE) described by generalized gradient approximation (GGA).[3] An all electron double numerical atomic orbital augmented by Double Numerical plus polarization (DNP) is used as the basis set, and the core treatment was all electron; The convergence criteria in total energy, maximum force, and maximum displacement were set at 10^{-5} Hartree, 0.002 Hartree/Å, and 0.005 Å, respectively. The electronic self-consistent field (SCF) tolerance was set at 10^{-6} Hartree.

Dipole moment: The structure was optimized using BP86 functional combined with SDD and 6-31G*, and the wave function was generated using B3LYP combined with SDD and 6-311G* in the water environment described by the IEFPCM model. The atomic charge and dipole moment were calculated using Multiwfn.[4]

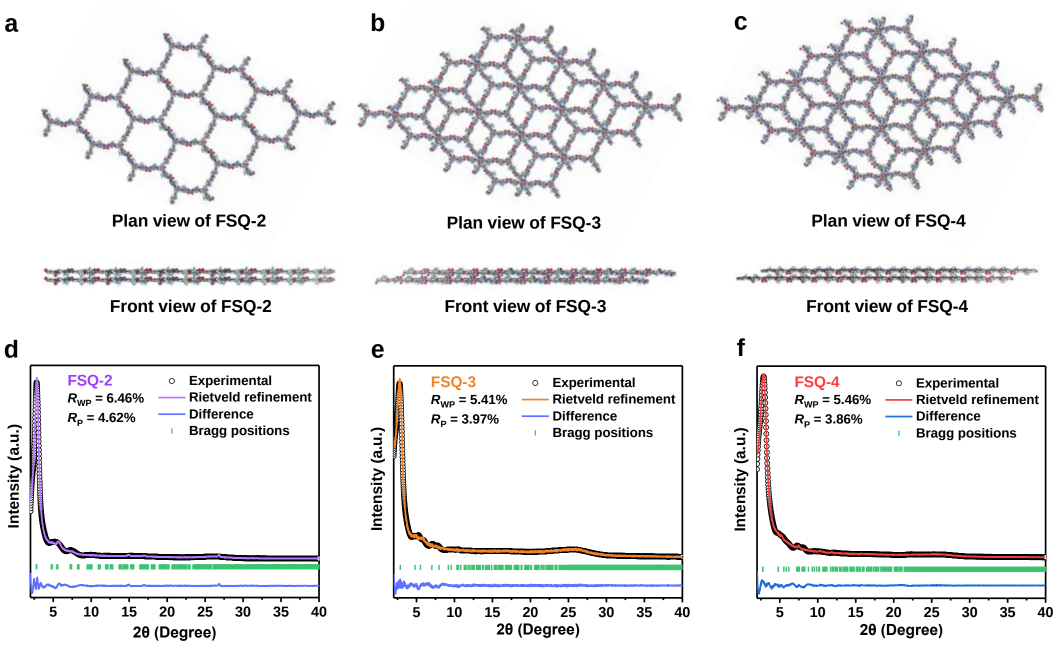

Figure S1. Simulated structures of (a) FSQ-2, (b) FSQ-3, and (c) FSQ-4. PXRD patterns of (d) FSQ-2, (e) FSQ-3, and (f) FSQ-4 with the experimental profiles and Rietveld refinement.

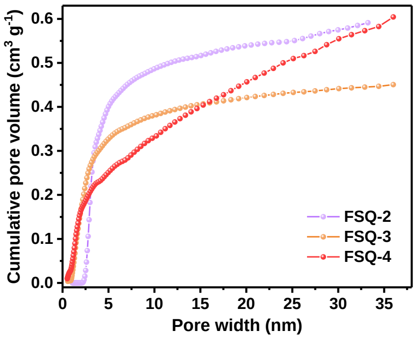

Figure S2. The cumulative pore volume of FSQ-2, FSQ-3, and FSQ-4.

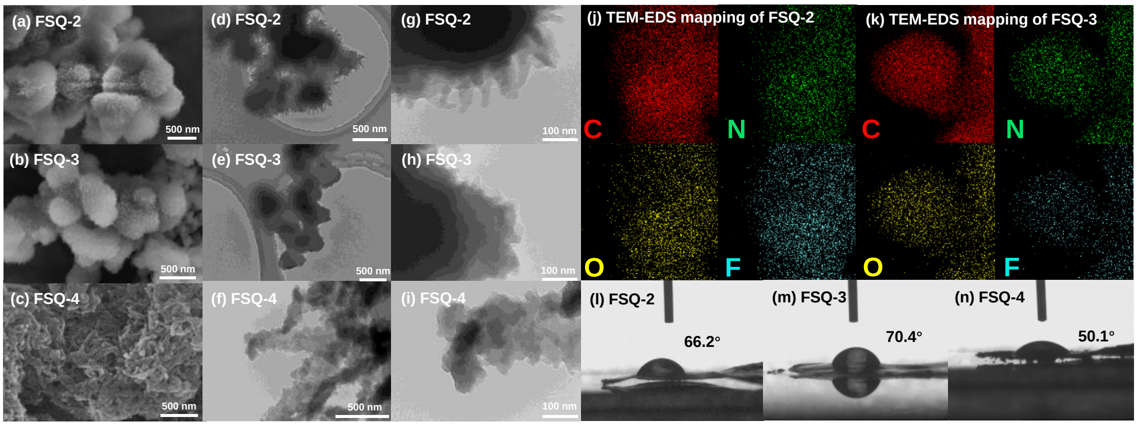

Figure S3. SEM images of (a) FSQ-2, (b) FSQ-3, and (c) FSQ-4. TEM images of (d, g) FSQ-2, (e, h) FSQ-3, and (f, i) FSQ-4 from Hitachi HT7800 TEM. TEM-EDS mappings of (j) FSQ-2 and (k) FSQ-3 from JEOL2010 spherical aberration corrected TEM. The water contact angle of (l) FSQ-2, (m) FSQ-3, and (n) FSQ-4.

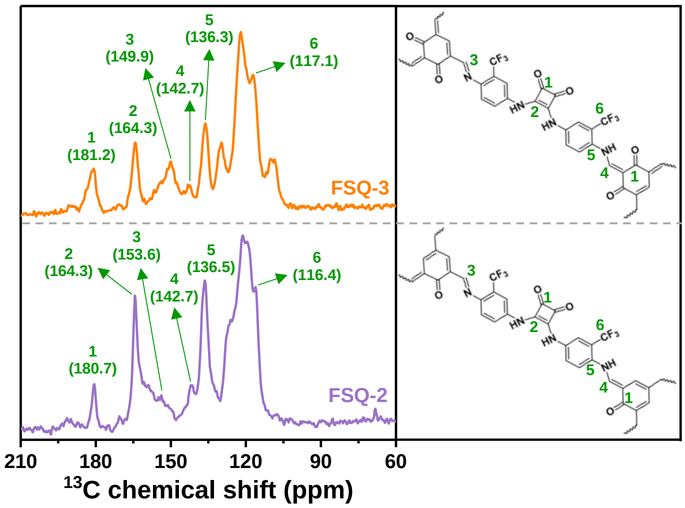

Figure S4. ^{13}C solid-state NMR spectra of FSQ-2 and FSQ-3.

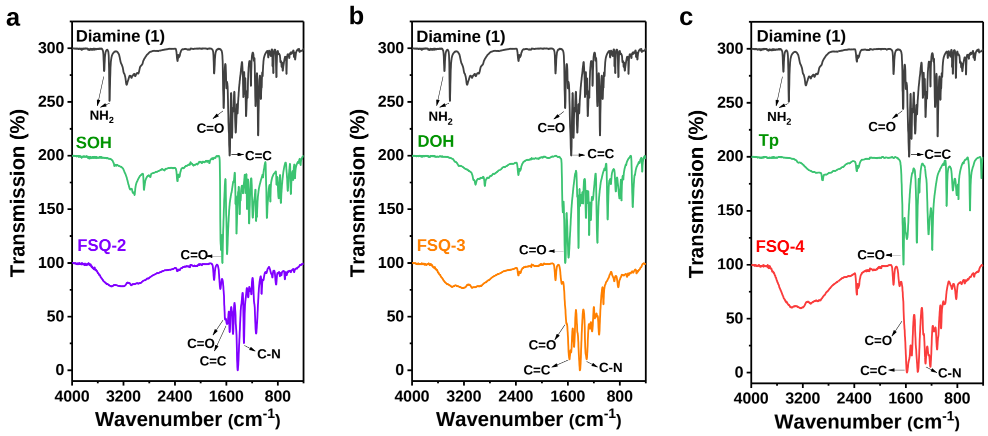

Figure S5. FT-IR spectra of (a) FSQ-2, (b) FSQ-3, and (c) FSQ-4.

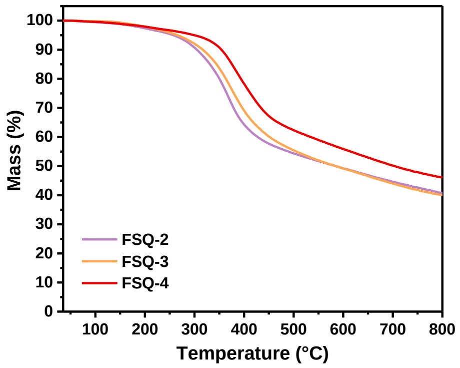

Figure S6. TGA curves of FSQ-2, FSQ-3, and FSQ-4.

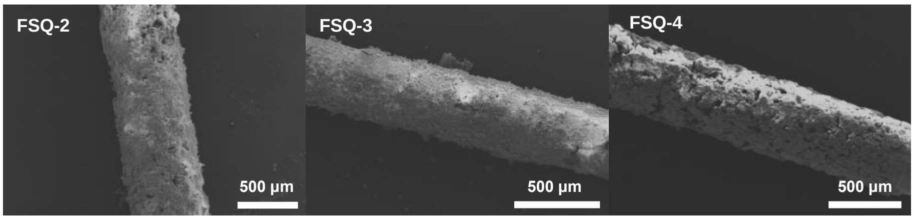

Figure S7. SEM images of FSQ-2, FSQ-3, and FSQ-4 SPME fibers.

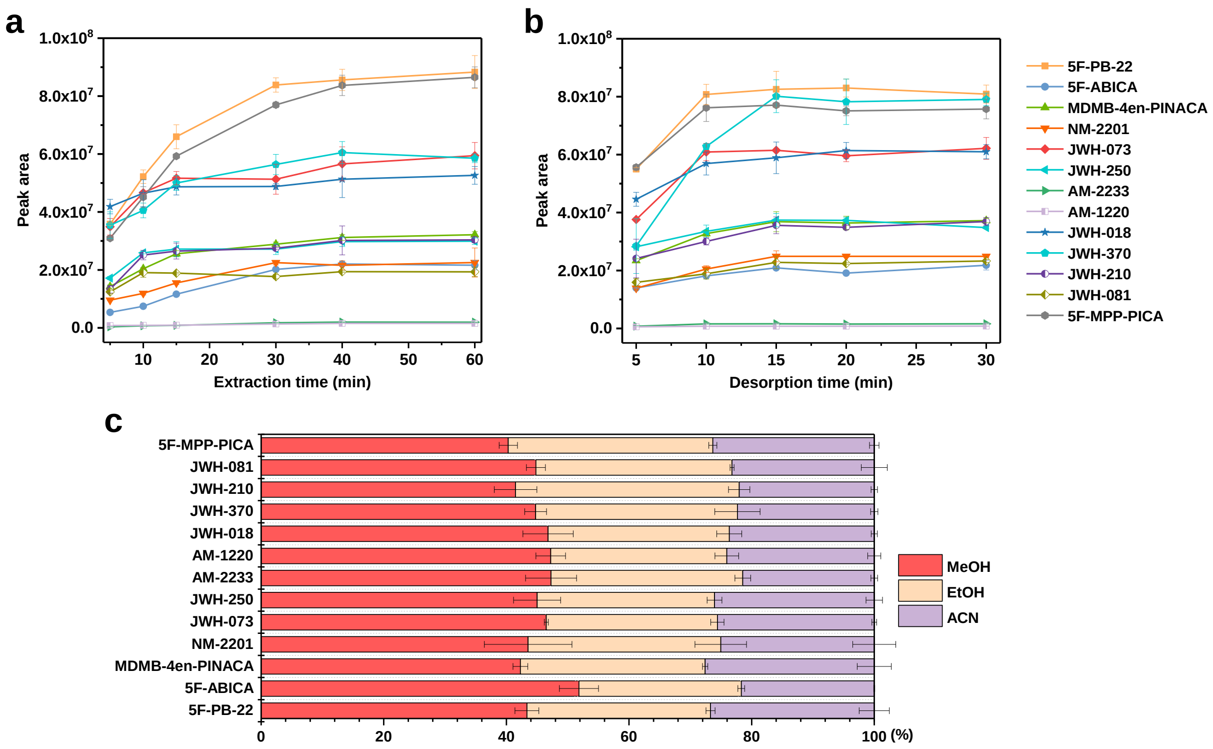

Figure S8. Effects of SPME conditions on the extraction efficiencies of FSQ-4 fiber toward SCs. (a) Extraction time, (b) desorption time, and (c) desorption solvent.

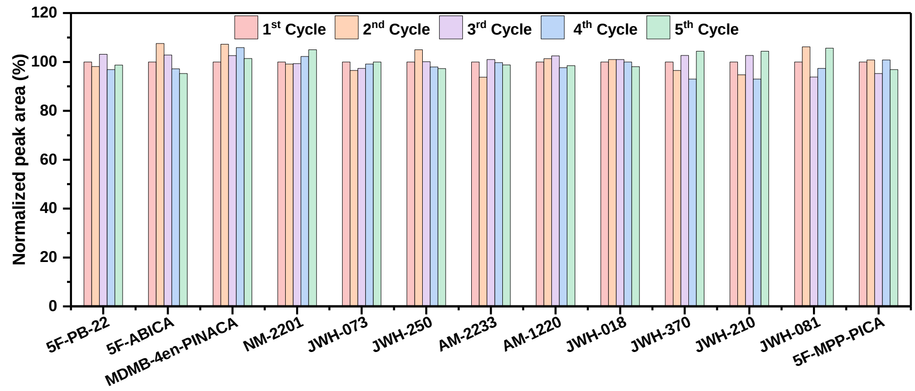

Figure S9. Multi-cycle extraction experiments to 13 SCs by FSQ-4 (the spent FSQ-4 fiber was regenerated by MeOH solution, V = 20 mL, 450 rpm, 30 min).

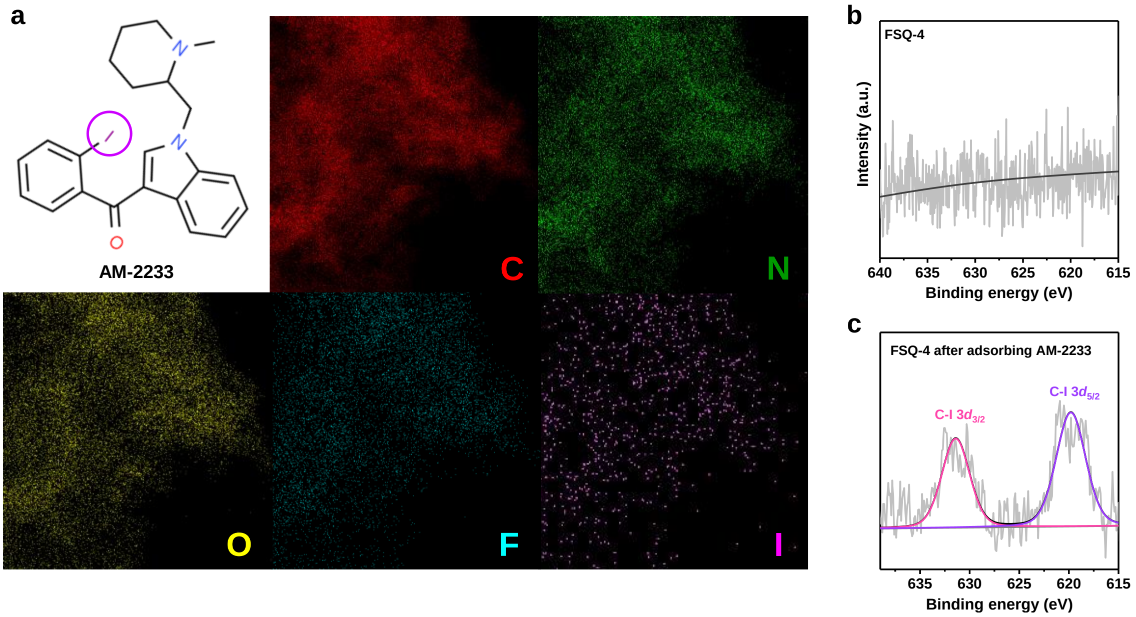

Figure S10. (a) TEM-EDS mappings of FSQ-4 after adsorbing AM-2233. I 3d XPS spectra of (b) FSQ-4 and (c) FSQ-4 after adsorbing AM-2233.

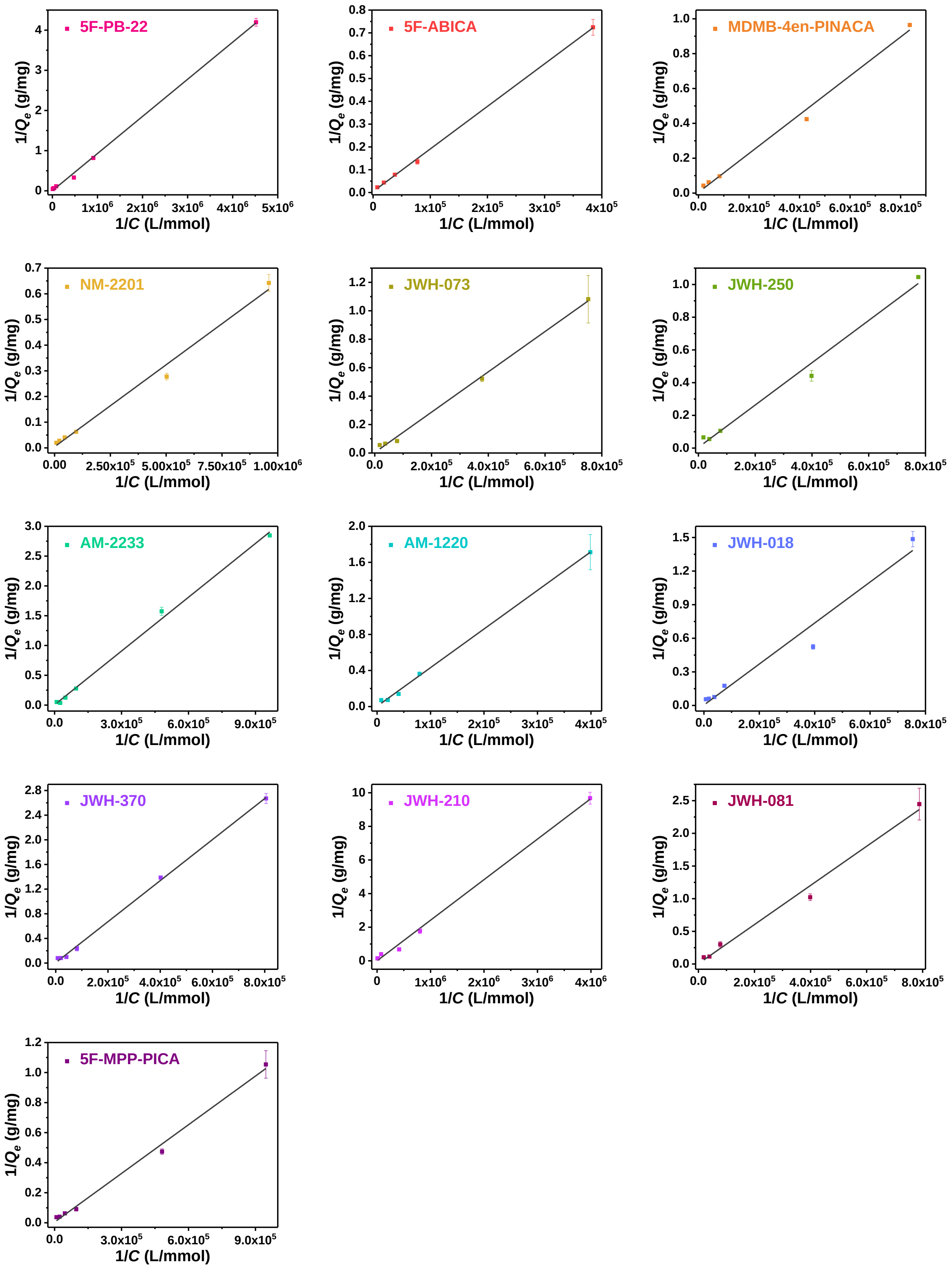

Figure S11. Thermodynamic profiles of 13 SCs adsorbed by FSQ-4 with Langmuir fitting.

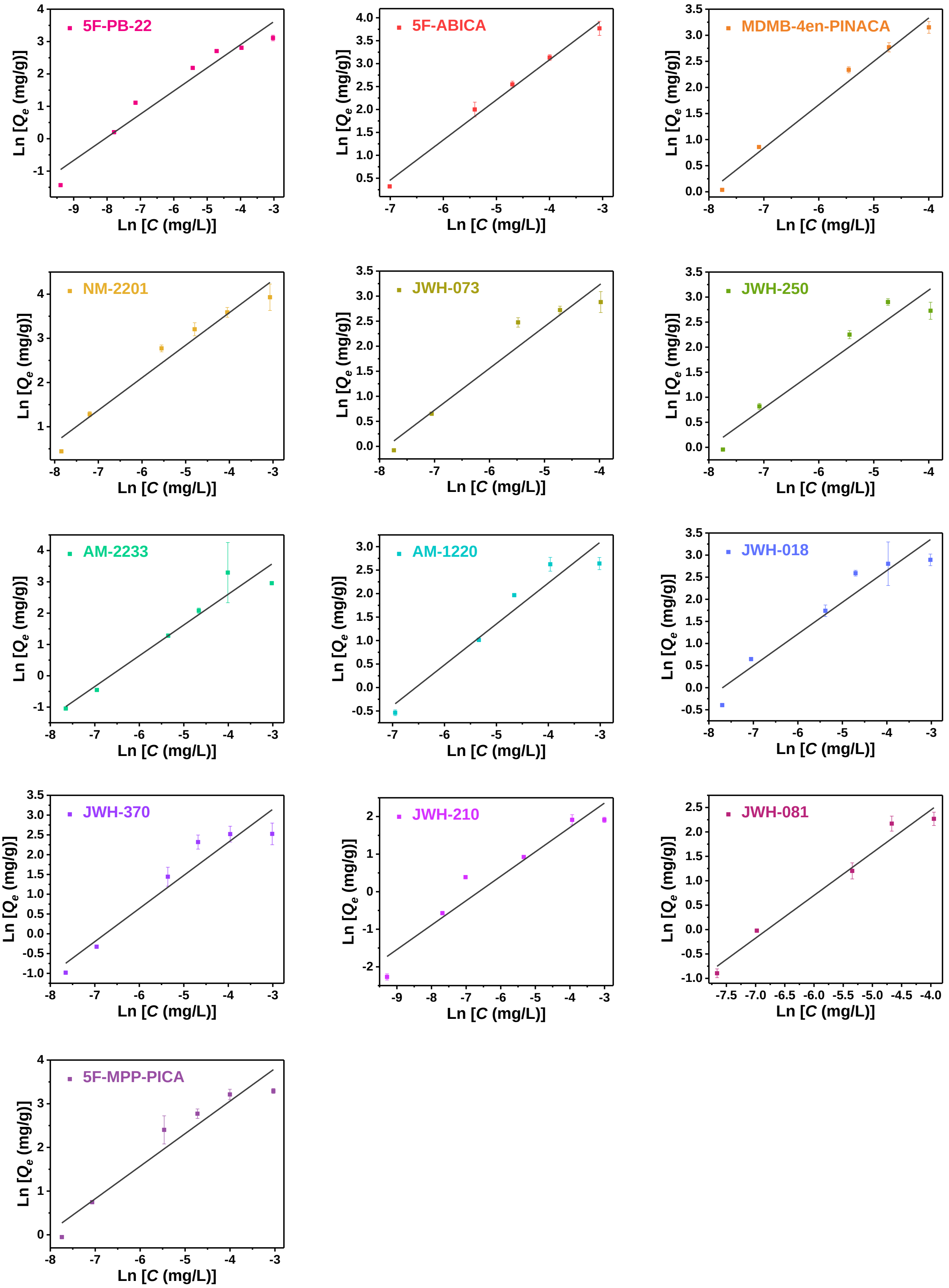

Figure S12. Thermodynamic profiles of 13 SCs adsorbed by FSQ-4 with Freundlich fitting.

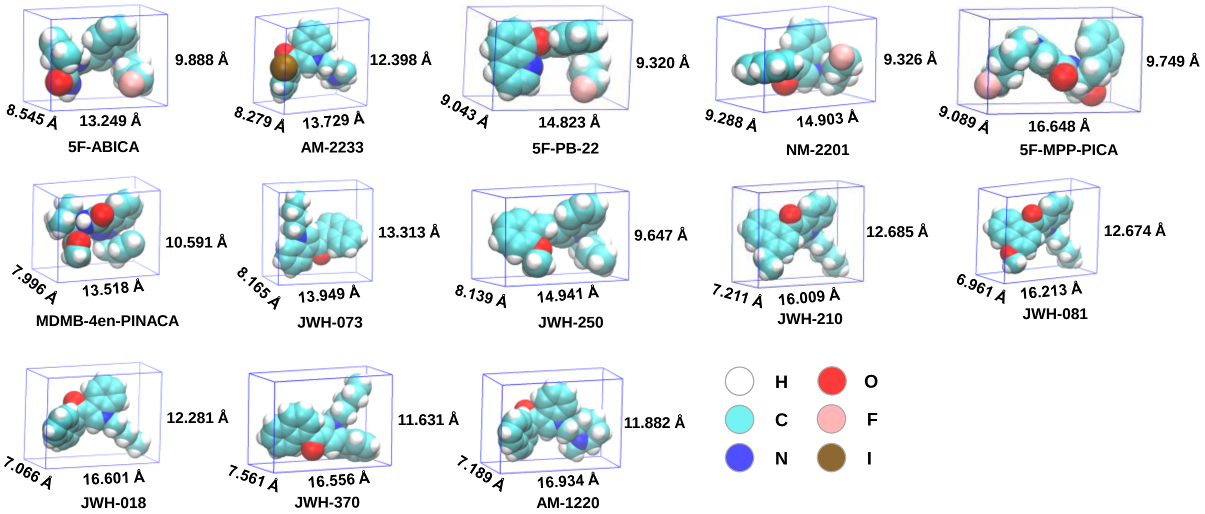

Figure S13. Molecular dimensions of the target SCs.

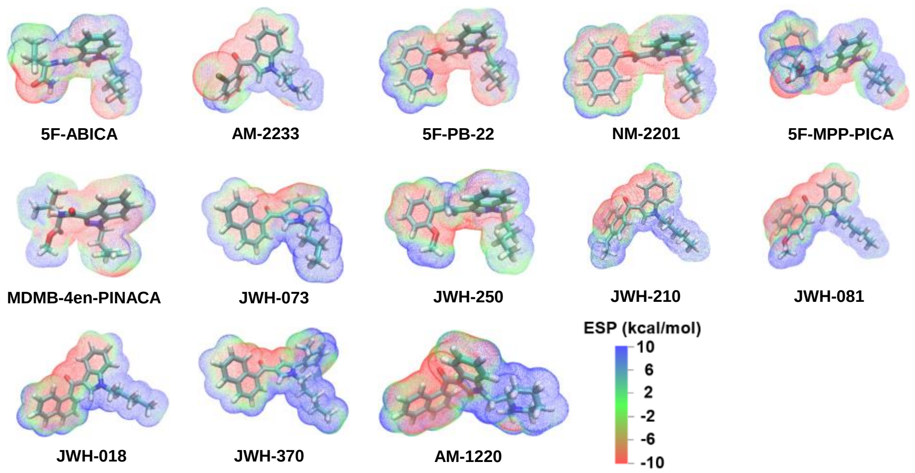

Figure S14. The surface electrostatic potential of SCs.

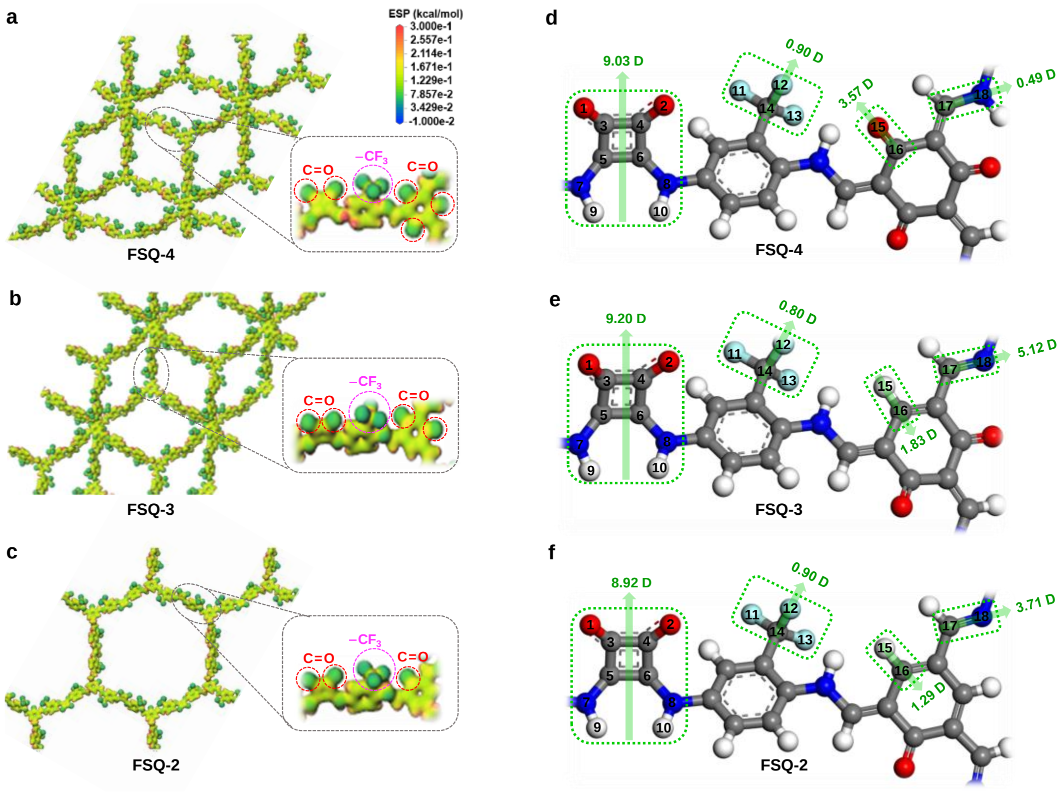

Figure S15. The surface electrostatic potential of (a) FSQ-4, (b) FSQ-3, and (c) FSQ-2. The dipole moment of (d) FSQ-4, (e) FSQ-3, and (f) FSQ-2.

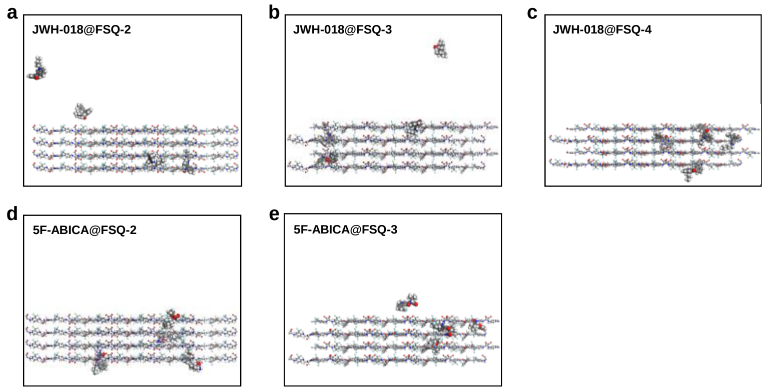

Figure S16. Model of (a) JWH-018@FSQ-2, (b) JWH-018@FSQ-3, (c) JWH-018@FSQ-4, (d) 5F-ABICA@FSQ-2, and (e) 5F-ABICA@FSQ-3 for MD simulation.

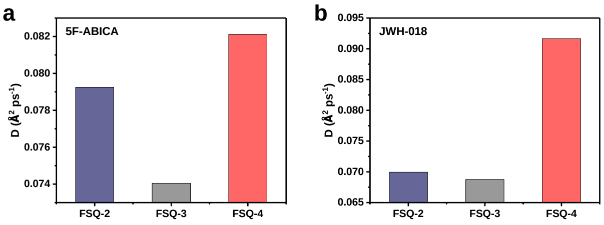

Figure S17. The diffusion coefficient of (a) 5F-ABICA and (b) JWH-018 in FSQ-2, FSQ-3, and FSQ-4, respectively.

Table S1. Reaction conditions that screened for COFs synthesis.

COFs	Temperature (°C)	Time (days)	HAc concentration (M)	HAc content (drops)	Crystallinity
FSQ-2	120	3	6	10	Yes
	120	3	9	10	Partial
	120	3	12	10	Yes
	120	3	17.5	10	Yes
	60	22	12	10	Yes
	90	22	12	10	Yes
	120	22	12	10	Yes
FSQ-3	120	3	6	10	Yes
	120	3	9	10	Partial
	120	3	12	10	No
	120	3	17.5	10	No
	60	22	12	10	No
	90	22	12	10	No
	120	22	12	10	Partial
FSQ-4	120	3	6	10	Yes
	120	3	9	10	Partial
	120	3	12	10	Yes
	120	3	17.5	10	Partial
	60	22	12	10	No
	90	22	12	10	No
	120	22	12	10	No

Table S2. Atomic coordinates of FSQ-2.

Space group: P1<br>a = b = 38.6785 Å, c = 4.1839 Å<br>α = β = 90°, γ = 120°											
	X	Y	Z		X	Y	Z		X	Y	Z
C	0.9355	0.7701	0.19443	C	0.2744	0.87073	-0.22717	C	0.08929	0.8224	0.03807
C	0.8939	0.74567	0.21545	C	0.43755	0.69082	-0.33488	C	0.06465	0.84198	0.04054
C	0.86824	0.75855	0.09811	F	0.24941	0.87693	-0.4247	C	0.08698	0.88636	0.04127
C	0.88543	0.79662	-0.04133	F	0.28793	0.89911	0.01199	C	0.12748	0.90801	0.03872
C	0.92733	0.82157	-0.06909	F	0.30692	0.87607	-0.40453	C	0.15157	0.88712	0.04253
C	0.95309	0.80822	0.05196	F	0.44173	0.72327	-0.50696	C	0.02395	0.81901	0.03715
N	0.82525	0.73554	0.14102	F	0.44364	0.66667	-0.54223	C	0.14591	0.9526	0.0162
C	0.80071	0.69401	0.05527	F	0.46686	0.70474	-0.10286	H	0.95387	0.7595	0.30261
C	0.75831	0.67107	0.0224	N	0.40741	0.61041	-0.04537	H	0.88232	0.71761	0.33766
C	0.75861	0.6391	0.1665	C	0.39363	0.56798	-0.0229	H	0.86564	0.80687	-0.12593
C	0.79877	0.66097	0.20403	C	0.41863	0.55256	0.0015	H	0.81043	0.75181	0.15862
O	0.82119	0.6574	0.40675	C	0.46274	0.57819	0.0379	H	0.7394	0.71327	0.11879
O	0.73225	0.60966	0.32457	C	0.48758	0.5632	0.02751	H	0.68779	0.60893	-0.19055
N	0.7289	0.68313	0.07063	C	0.47109	0.5193	-0.00631	H	0.60106	0.65459	0.29998
C	0.68598	0.65638	0.06127	C	0.43143	0.49282	-0.01814	H	0.67316	0.69739	0.31174
C	0.66856	0.61858	-0.08503	C	0.40291	0.50841	-0.01125	H	0.54121	0.62289	0.08757
C	0.62656	0.59321	-0.09698	O	0.36703	0.4852	-0.02035	H	0.16746	0.76688	0.33518
C	0.60122	0.60625	0.03511	C	0.24216	0.0418	-0.10273	H	0.19761	0.72477	0.32439
C	0.61901	0.64401	0.18456	C	0.26403	0.08355	-0.13546	H	0.29767	0.81438	-0.20079
C	0.6606	0.66862	0.19504	C	0.24809	0.10742	-0.03887	H	0.24699	0.70933	0.1053
C	0.94382	0.86242	-0.22143	C	0.20954	0.08816	0.09369	H	0.28159	0.66905	0.0892
C	0.60893	0.55209	-0.24584	C	0.18687	0.04603	0.13235	H	0.38355	0.71516	-0.26071
F	0.95826	0.89155	0.01197	C	0.20335	0.02188	0.02568	H	0.33304	0.58083	0.20923
F	0.9745	0.86971	-0.42835	N	0.2719	0.1508	-0.06512	H	0.29293	0.61355	0.23972
F	0.91478	0.86534	-0.39307	C	0.25695	0.17769	-0.00032	H	0.43774	0.62961	-0.07747
F	0.57785	0.54512	-0.44755	C	0.27647	0.22035	0.00238	H	0.36186	0.54745	-0.04264
F	0.63713	0.54807	-0.4203	C	0.24289	0.21768	-0.1391	H	0.25566	0.02457	-0.18137
F	0.59438	0.52339	-0.00907	C	0.22442	0.17721	-0.14279	H	0.29373	0.09734	-0.23691
N	0.55809	0.58045	0.01968	O	0.19682	0.15165	-0.32029	H	0.19721	0.10628	0.17353
C	0.53155	0.59162	0.05375	O	0.23809	0.24193	-0.31227	H	0.30219	0.1636	-0.12023
C	0.19552	0.77777	0.21047	N	0.31746	0.25027	-0.06036	H	0.33734	0.24023	-0.11407
C	0.213	0.75362	0.20797	C	0.33276	0.29319	-0.0483	H	0.28246	0.28976	0.19913
C	0.24985	0.76621	0.05914	C	0.31133	0.30964	0.09612	H	0.41502	0.37998	-0.27606
C	0.26929	0.80434	-0.08427	C	0.32683	0.35152	0.10943	H	0.38759	0.30794	-0.29178
C	0.25211	0.82938	-0.08148	C	0.36513	0.37792	-0.01985	H	0.44094	0.441	-0.07211
C	0.21403	0.81548	0.05949	C	0.38635	0.36119	-0.16512	H	0.13773	0.79168	0.02374
N	0.26604	0.7394	0.05046	C	0.37045	0.31962	-0.1774	H	0.01111	0.78699	0.02285
C	0.30657	0.75113	-0.03419	C	0.14656	0.02963	0.29409	H	0.1257	0.96331	-0.03571
C	0.32611	0.72811	-0.0477	C	0.30216	0.36789	0.25733	N	0.99634	0.83397	0.04648
C	0.35934	0.76042	0.0854	F	0.11696	0.01892	0.0674	N	0.18391	-0.0217	0.04994
C	0.34078	0.78234	0.09708	F	0.13823	-0.00283	0.48023	H	1.0076	0.86464	0.04778
O	0.34615	0.81091	0.27889	F	0.14576	0.05785	0.49273	H	0.49206	0.50846	-0.01368
O	0.38808	0.76271	0.24881	F	0.28731	0.38166	0.02008	O	0.07296	0.78601	0.03556
N	0.31147	0.68643	0.02159	F	0.32546	0.39924	0.45966	H	0.18383	0.90498	0.04272
C	0.33552	0.66721	-0.00538	F	0.27034	0.339	0.43104	H	0.07056	0.90216	0.03713
C	0.37281	0.68591	-0.1589	N	0.38085	0.42092	-0.00746	H	0.47583	0.60981	0.07942
C	0.39663	0.66765	-0.17989	C	0.41808	0.44904	-0.04108				
C	0.38233	0.62872	-0.04827	N	0.19462	0.83955	0.05005				
C	0.3445	0.60983	0.0993	C	0.15587	0.82382	0.04006				
C	0.32155	0.62876	0.11987	C	0.13376	0.84665	0.04064				

Table S3. Atomic coordinates of FSQ-3.

Space group: P1
a = b = 38.6793 Å, c = 4.1839 Å
α = β = 90°, γ = 120°

	X	Y	Z		X	Y	Z		X	Y	Z
C	0.95072	0.78919	0.18806	C	0.2927	0.89673	-0.22641	C	0.15031	0.8724	0.08439
C	0.90902	0.7666	0.20977	C	0.4535	0.71845	-0.33761	C	0.10598	0.84914	0.06471
C	0.88482	0.78044	0.07784	F	0.26898	0.90408	-0.42885	C	0.08273	0.8658	0.04859
C	0.90362	0.81817	-0.0692	F	0.30706	0.92532	0.01121	C	0.10147	0.91011	0.03468
C	0.94573	0.84152	-0.09197	F	0.32493	0.90069	-0.40039	C	0.14578	0.9369	0.07378
C	0.96976	0.82669	0.03528	F	0.45753	0.75122	-0.50375	C	0.1694	0.91647	0.13938
N	0.84173	0.75874	0.11364	F	0.45938	0.6947	-0.55078	O	0.20228	0.93459	0.2622
C	0.81646	0.71764	0.0193	F	0.48305	0.73193	-0.1078	O	0.08016	0.92462	-0.00815
C	0.77412	0.69544	-0.01575	N	0.42397	0.63785	-0.05231	C	0.03832	0.83835	0.04711
C	0.77404	0.66248	0.11273	C	0.40967	0.59545	-0.02777	C	0.16124	0.97737	0.05157
C	0.8142	0.68357	0.15056	C	0.43409	0.57969	0.01637	H	0.96796	0.77778	0.30407
O	0.83645	0.67818	0.34271	C	0.4773	0.60428	0.09694	H	0.89627	0.73936	0.34469
O	0.74743	0.63214	0.26087	C	0.50439	0.58777	0.04708	H	0.88506	0.82945	-0.1625
N	0.74476	0.70739	0.04023	C	0.48539	0.54347	0.01738	H	0.8278	0.77589	0.12773
C	0.70192	0.68048	0.0217	C	0.44546	0.51874	-0.00403	H	0.75514	0.73718	0.10089
C	0.68549	0.64341	-0.13417	C	0.41804	0.53564	-0.01198	H	0.70545	0.63513	-0.24583
C	0.64378	0.61679	-0.14486	O	0.49017	0.63676	0.2252	H	0.61564	0.67509	0.26956
C	0.61737	0.62825	-0.00429	O	0.38225	0.5132	-0.04601	H	0.68755	0.71968	0.28283
C	0.63426	0.66576	0.14816	C	0.25657	0.07185	-0.1179	H	0.56455	0.57098	-0.03221
C	0.67574	0.69138	0.15966	C	0.27648	0.11341	-0.15529	H	0.55522	0.64489	0.05147
C	0.96483	0.88267	-0.24191	C	0.26006	0.13598	-0.03895	H	0.18317	0.79432	0.33348
C	0.62836	0.57602	-0.29735	C	0.22272	0.11564	0.1134	H	0.21189	0.75082	0.32475
F	0.98039	0.91144	-0.00599	C	0.20198	0.07355	0.15675	H	0.31311	0.83888	-0.19735
F	0.99545	0.88826	-0.44373	C	0.2193	0.05079	0.03688	H	0.26168	0.73465	0.11585
F	0.93742	0.88792	-0.41682	N	0.28236	0.17926	-0.06722	H	0.29705	0.69467	0.09497
F	0.59708	0.5675	-0.50096	C	0.26669	0.205	0.01517	H	0.39885	0.74193	-0.259
F	0.65773	0.57405	-0.47209	C	0.28578	0.24752	0.01635	H	0.35017	0.60794	0.21068
F	0.61516	0.54702	-0.06312	C	0.25177	0.24465	-0.11808	H	0.30944	0.63989	0.2455
N	0.57433	0.6011	-0.00235	C	0.23363	0.20419	-0.12008	H	0.45416	0.65632	-0.09981
C	0.54471	0.61325	0.0372	O	0.20589	0.17844	-0.29552	H	0.37819	0.57526	-0.06928
C	0.21124	0.80461	0.21047	O	0.24657	0.26854	-0.29267	H	0.27065	0.05575	-0.21022
C	0.22786	0.77966	0.20884	N	0.32647	0.27702	-0.05806	H	0.30517	0.12811	-0.27444
C	0.26477	0.79163	0.0632	C	0.34229	0.31991	-0.0457	H	0.20989	0.13289	0.20411
C	0.28484	0.82957	-0.08139	C	0.32155	0.33687	0.10231	H	0.31217	0.19291	-0.13977
C	0.26879	0.85549	-0.07933	C	0.33771	0.37882	0.11549	H	0.3459	0.26665	-0.11845
C	0.23077	0.84251	0.06347	C	0.37601	0.40459	-0.01584	H	0.29274	0.31736	0.20809
N	0.28076	0.76467	0.05971	C	0.39643	0.38732	-0.16579	H	0.42496	0.40572	-0.28009
C	0.32139	0.77661	-0.02344	C	0.37985	0.34576	-0.17885	H	0.39637	0.3336	-0.29664
C	0.34121	0.75396	-0.0392	C	0.1624	0.05544	0.33156	H	0.45242	0.46529	-0.049
C	0.37438	0.78632	0.094	C	0.31375	0.3959	0.26478	H	0.23092	0.89837	0.04188
C	0.35551	0.80791	0.10831	F	0.13152	0.04266	0.11404	H	0.15171	0.81929	0.0275
O	0.36075	0.83638	0.29082	F	0.15692	0.02394	0.51857	H	0.02726	0.80658	0.0621
O	0.40335	0.7888	0.25542	F	0.16063	0.08306	0.53126	H	0.14051	0.98748	0.00199
N	0.32689	0.71233	0.02736	F	0.29922	0.41011	0.02826	N	1.01298	0.85119	0.02533
C	0.35137	0.69365	-0.00172	F	0.33757	0.42711	0.46649	N	0.20287	0.00786	0.08293
C	0.38853	0.71275	-0.15733	F	0.28175	0.36739	0.43938	H	0.224	-0.00131	0.08405
C	0.41274	0.69496	-0.18072	N	0.39272	0.44766	-0.00139	H	0.50391	0.52993	0.02125
C	0.39888	0.6561	-0.04981	C	0.43043	0.47466	-0.02637	H	0.09167	0.81681	0.06731
C	0.36124	0.6369	0.10088	N	0.21256	0.86766	0.06574				
C	0.33793	0.65536	0.12383	C	0.16979	0.85124	0.06299				

Table S4. Atomic coordinates of FSQ-4.

Space group: P1											
a = b = 38.6801 Å, c = 4.1839 Å											
α = β = 90°, γ = 120°											
	X	Y	Z		X	Y	Z		X	Y	Z
C	0.93665	0.75098	0.18469	C	0.27933	0.85484	-0.25053	C	0.15589	0.80749	0.01121
C	0.89504	0.72698	0.20876	C	0.44155	0.67678	-0.34468	C	0.135	0.8274	0.03034
C	0.8696	0.74062	0.10079	F	0.25539	0.86178	-0.45376	C	0.09061	0.80362	-0.00903
C	0.88704	0.779	-0.03316	F	0.29341	0.88352	-0.01365	C	0.06573	0.82307	-0.01333
C	0.92894	0.80347	-0.0646	F	0.3117	0.85904	-0.42389	C	0.08718	0.86705	-0.06349
C	0.95442	0.78939	0.0479	F	0.44573	0.70948	-0.51302	C	0.13058	0.89248	0.02061
N	0.8266	0.71781	0.14468	F	0.44756	0.65288	-0.55524	C	0.15299	0.87111	0.10255
C	0.80198	0.67632	0.05862	F	0.47087	0.69035	-0.11297	O	0.1842	0.8883	0.25097
C	0.75967	0.65346	0.02002	N	0.41153	0.5961	-0.06059	O	0.06989	0.88223	-0.19537
C	0.76019	0.6214	0.16073	C	0.39686	0.55353	-0.05648	O	0.07422	0.76734	-0.03588
C	0.80024	0.64318	0.20345	C	0.42047	0.53701	-0.00239	C	0.02513	0.79986	0.01138
O	0.82224	0.6394	0.40911	C	0.46249	0.56062	0.11496	C	0.14651	0.93312	0.01854
O	0.73364	0.59124	0.31091	C	0.48983	0.54418	0.09029	H	0.95486	0.7398	0.2862
N	0.72985	0.66499	0.06878	C	0.47087	0.49992	0.11637	H	0.88336	0.69859	0.3256
C	0.68707	0.63763	0.05058	C	0.4276	0.47333	0.03598	H	0.86749	0.78984	-0.11143
C	0.67103	0.60045	-0.10275	C	0.40448	0.49333	-0.06315	H	0.81186	0.73415	0.16356
C	0.62934	0.57368	-0.11793	O	0.47411	0.59251	0.24991	H	0.73982	0.69482	0.125
C	0.60258	0.58494	0.01915	O	0.37342	0.47459	-0.21221	H	0.69129	0.59224	-0.20942
C	0.61912	0.62245	0.17145	O	0.48995	0.48537	0.22617	H	0.60018	0.63164	0.28953
C	0.66054	0.64832	0.18493	C	0.24265	0.02741	-0.12101	H	0.67204	0.67669	0.30647
C	0.9458	0.84453	-0.21339	C	0.26271	0.06905	-0.15669	H	0.54927	0.52799	-0.03649
C	0.61434	0.53309	-0.27267	C	0.24588	0.09157	-0.0471	H	0.54008	0.60149	0.05185
F	0.96005	0.87334	0.02248	C	0.20802	0.07106	0.09697	H	0.16982	0.75191	0.30394
F	0.97673	0.85196	-0.41809	C	0.18716	0.02896	0.13849	H	0.19911	0.70885	0.29996
F	0.91705	0.84778	-0.3864	C	0.20481	0.00623	0.02522	H	0.30062	0.79758	-0.21555
F	0.58396	0.52507	-0.48467	N	0.26821	0.13494	-0.07269	H	0.24924	0.69309	0.09444
F	0.64426	0.53116	-0.43926	C	0.252	0.16042	0.0031	H	0.28459	0.65305	0.07399
F	0.60005	0.50372	-0.04119	C	0.27037	0.20291	0.01026	H	0.38707	0.7006	-0.26869
N	0.55951	0.55777	0.01725	C	0.23618	0.19935	-0.12678	H	0.33726	0.56587	0.1906
C	0.52994	0.56987	0.06134	C	0.21874	0.15896	-0.13418	H	0.29654	0.59796	0.22298
C	0.19813	0.76243	0.18416	O	0.19147	0.13316	-0.3126	H	0.44201	0.61475	-0.09542
C	0.21506	0.73775	0.18488	O	0.23031	0.22323	-0.29563	H	0.36589	0.53403	-0.12102
C	0.25217	0.74999	0.04178	N	0.31097	0.23366	-0.05389	H	0.25703	0.01136	-0.20797
C	0.27218	0.78801	-0.10188	C	0.3252	0.27627	-0.03145	H	0.29182	0.08385	-0.26909
C	0.25575	0.81364	-0.10247	C	0.30263	0.29114	0.1169	H	0.19486	0.08817	0.18367
C	0.21753	0.80033	0.03734	C	0.31664	0.33266	0.13829	H	0.29833	0.14879	-0.13644
N	0.26831	0.72314	0.03909	C	0.35491	0.36041	0.01187	H	0.33139	0.22452	-0.11376
C	0.30901	0.73511	-0.0421	C	0.3774	0.34521	-0.13389	H	0.27389	0.27019	0.21621
C	0.32889	0.71246	-0.05592	C	0.36282	0.30393	-0.15434	H	0.4061	0.36533	-0.23993
C	0.36192	0.74478	0.07919	C	0.14717	0.01085	0.30673	H	0.38089	0.29346	-0.27067
C	0.34301	0.76634	0.09159	C	0.29022	0.34661	0.29279	H	0.34912	0.41279	0.03538
O	0.34809	0.79479	0.2737	F	0.11688	-0.00102	0.08477	H	0.43159	0.42144	0.10977
O	0.3907	0.7472	0.24297	F	0.14078	-0.02128	0.48899	H	0.21666	0.8558	0.01879
N	0.31455	0.67078	0.00948	F	0.14536	0.03818	0.50954	H	0.1393	0.77576	-0.03753
C	0.33904	0.65207	-0.01743	F	0.2741	0.35976	0.06086	H	0.01247	0.76783	0.01819
C	0.3765	0.67129	-0.16867	F	0.31216	0.37756	0.50181	H	0.12627	0.94382	-0.03161
C	0.40064	0.6534	-0.19024	F	0.25915	0.31625	0.46256	N	0.99765	0.81482	0.04033
C	0.38649	0.6144	-0.06161	N	0.3699	0.40329	0.0187	N	0.18794	-0.03683	0.06931
C	0.34856	0.59504	0.08387	C	0.41179	0.43275	0.05158	H	1.00905	0.84528	0.07384
C	0.32528	0.61357	0.10516	N	0.1987	0.82493	0.03464	H	0.20882	-0.04631	0.0793

Table S5. The peak areas of C–N and C=O in FSQ-2, FSQ-3, and FSQ-4.

	C–N (286.1 eV)		C=O (288.5 eV)	
	Peak areas	Area ratio (%)	Peak areas	Area ratio (%)
FSQ-2	9872.65	72.17	3426.03	66.76
FSQ-3	12431.40	90.87	4780.88	93.16
FSQ-4	13679.85	100.00	5131.72	100.00

Table S6. The physical properties of synthetic cannabinoids were investigated in this work.

Cas number	Analytes	Structure	Molecular weight	logP [a)]	LogD (pH 7.4)	pKa [a)]	Halogen atom	H-bond acceptors [a)]	H-bond donors [a)]
2042201-16-9	NM-2201	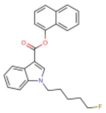	375.44	6.169	6.17	-	1 (F)	3	0
1801338-26-0	5F-ABICA	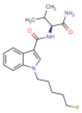	347.43	2.720	2.74	15.15	1 (F)	5	3
1400742-41-7	5F-PB-22	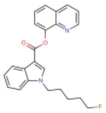	376.43	5.338	5.34	3.01	1 (F)	4	0
2504100-70-1	MDMB-4en-PINACA	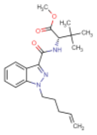	357.45	3.803	3.80	14.65	0	6	1
208987-48-8	JWH-073	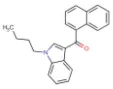	327.42	6.068	6.07	-	0	2	0
864445-43-2	JWH-250	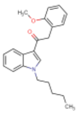	335.45	5.298	5.30	13.66	0	3	0
444912-75-8	AM-2233	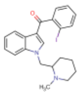	458.34	5.613	3.82	9.2	1 (I)	3	0
137642-54-7	AM-1220	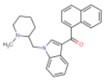	382.50	5.673	3.88	9.2	0	3	0
209414-07-3	JWH-018	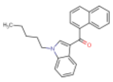	341.45	6.513	6.51	-	0	2	0
914458-22-3	JWH-370	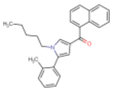	381.51	7.575	7.58	-	0	2	0
824959-81-1	JWH-210	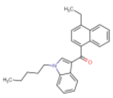	369.50	7.471	7.47	-	0	2	0

210179-46-7	JWH-081	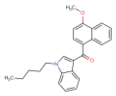	371.47	6.355	6.95	-	0	3	0
-	5F-MPP-PICA	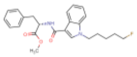	410.48	4.442	4.24	-	1 (F)	5	1

[a] Statistics were obtained from ChemSpider (http://www.chemspider.com/) and ChemAxon (https://chemaxon.com/).

Table S7. The comparison of LODs (ng/L) between the present method and others in reported works.

Ref.	Online	Analyzation technique	Sample preparation method	5F-PB-22	5F-ABICA	MDMB-4en-PINACA	NM-2201	JWH-073	JWH-250	AM-2233	AM-1220	JWH-018	JWH-370	JWH-210	JWH-081	5F-MPP-PICA
5	2023	LC-FLD	SPE	700	-	700	-	-	-	-	-	-	-	-	-	-
6	2022	LC-MS/MS	SPE		0.005	0.005	-	-	-	-	-	-	-	-	-	-
7	2020	UPLC-MS/MS	SPE	-	-	-	0.40	-	-	-	-	-	-	-	-	-
8	2023	LC-MS	Direct injection	-	-	0.20	-	-	-	-	-	-	-	-	-	-
9	2022	LC-HRMS	Direct injection	7500	5000	5000	-	5000	5000	5000	5000	5000	5000	5000	7500	5000
10	2022	UPLC-MS/MS	SLE	-	2500	-	2500	-	-	2500	2500	-	-	2500	2500	-
11	2021	LC-MS	LLE	25	-	-	-	50	-	-	-	100	-	-	-	-
12	2020	LC-MS/MS	Direct injection	-	-	-	-	-	-	-	-	75	-	-	-	-
13	2020	LC-MS/MS	SPE	5	-	-	-	5	-	-	-	1	-	-	-	-
14	2021	LC-MS/MS	DLLME	-	-	-	-	21	6	-	-	10	-	-	-	-
15	2019	SERS	μ-SPE	-	-	-	-	-	-	-	-	800	-	-	-	-
16	2018	HPLC-MS/MS	μ-SPE	-	-	-	-	32	360	80	-	442	-	318	624	-
17	2019	GC-HRMS	LLE	-	-	-	-	-	-	-	-	-	-	100000	-	-
18	2017	LC-MS/MS	μ-SPE	-	-	-	-	100	250	-	-	100	-	-	1000	-
19	2017	SERS	SLE	-	-	-	-	51000	-	-	-	18000	-	-	34000	-
**This work**		**UPLC-MS/MS**	**SPME**	**0.07**	**0.07**	**0.08**	**0.06**	**0.02**	**0.01**	**0.09**	**0.11**	**0.02**	**0.01**	**0.02**	**0.03**	**0.02**

Table S8. Original amounts and recoveries of SCs for wastewater samples.

Analytes	added (100 ng/L)			added (200 ng/L)			added (500 ng/L)		
	original amount (μg/L)	recovery (%)	RSD (%, n=3)	original amount (ng/L)	recovery (%)	RSD (%, n=3)	original amount (ng/L)	recovery (%)	RSD (%, n=3)
5F-PB-22	ND	87.3	2.5	ND	83.9	9.1	ND	99.1	4.4
5F-ABICA	ND	93.2	2.8	ND	80.5	9.6	ND	92.6	9.2
MDMB-4en-PINACA	ND	107.2	1.9	ND	100.7	8.2	ND	96.3	12.8
NM2201	ND	88.3	5.3	ND	89.6	6.7	ND	98.7	4.4
JWH-073	ND	102.8	3.5	ND	107.7	7.5	ND	84.1	4.7
JWH-250	ND	103.5	5.4	ND	88.8	2.2	ND	82.6	4.5
AM-2233	ND	109.0	6.1	ND	85.0	1.6	ND	108.8	9.1
AM-1220	ND	112.8	15.7	ND	102.8	9.6	ND	99.1	5.1
JWH-018	ND	98.9	9.3	ND	99.1	2.0	ND	103.6	6.2
JWH-370	ND	92.9	1.3	ND	103.2	1.6	ND	113.9	9.6
JWH-210	ND	89.4	6.3	ND	113.7	2.7	ND	110.0	6.8
JWH-081	ND	93.4	4.2	ND	110.5	6.7	ND	110.8	2.4
5F-MPP-PICA	ND	90.1	1.2	ND	90.0	7.9	ND	95.1	0.6

Table S9. Thermodynamic fitting results for 13 SCs on FSQ-4.

	Langmuir Model			Freundlich Model		
	Q_{max} (mg/g)	K_L (L/mmol)	R^2	1/n	K_F (mg/g)(L/mg)$^{1/n}$	R^2
5F-PB-22	270.5	4017.9	0.9987	0.71	318.0	0.9469
5F-ABICA	227.6	2349.8	0.9993	0.87	722.3	0.9906
MDMB-4en-PINACA	221.5	4067.1	0.9927	0.83	778.7	0.9831
NM2201	225.6	6924.8	0.9897	0.83	778.7	0.9614
JWH-073	278.7	2527.2	0.9971	0.83	703.4	0.9437
JWH-250	212.8	3642.3	0.9873	0.79	533.5	0.9338
AM-2233	221.2	1502.2	0.9966	0.98	691.6	0.9430
AM-1220	289.2	806.07	0.9984	0.87	302.4	0.9348
JWH-018	270.3	2021.7	0.9657	0.72	249.5	0.9304
JWH-370	171.3	1753.2	0.9986	0.84	286.5	0.9252
JWH-210	175.0	2370.6	0.9971	0.65	74.53	0.9268
JWH-081	201.6	1658.7	0.9890	0.87	382.8	0.9753
5F-MPP-PICA	219.7	4214.7	0.9947	0.75	421.1	0.9359

Table S10. Atomic charge of FSQ-4, FSQ-3, and FSQ-2.

Atomic	Atomic charge (*e*)		
(Figure S15)	FSQ-4	FSQ-3	FSQ-2
1	-0.280	-0.281	-0.280
2	-0.280	-0.280	-0.277
3	0.115	0.115	0.117
4	0.116	0.116	0.119
5	0.056	0.056	0.056
6	0.058	0.058	0.059
7	-0.037	-0.037	-0.035
8	-0.038	-0.038	-0.037
9	0.154	0.155	0.156
10	0.153	0.153	0.154
11	-0.097	-0.097	-0.094
12	-0.112	-0.111	-0.109
13	-0.110	-0.109	-0.108
14	0.301	0.302	0.303
15	-0.292	0.055	0.058
16	0.113	-0.005	-0.003
17	0.063	0.051	0.058
18	-0.038	-0.170	-0.156

Table S11. The mobile phase gradients for UPLC-MS/MS analysis. Solvent A referred to water with 0.1% formic acid, and Solvent B was ACN.

Time (min)	Flow rate (mL min^{-1})	Solvent A (%)	Solvent B (%)
Initial	0.400	95.0	5.0
8.00	0.400	10.0	90.0
9.50	0.400	10.0	90.0
9.60	0.400	95.0	5.0
12.00	0.400	95.0	5.0
17.00	0.400	95.0	5.0

Table S12. The chromatographic retention time of the SCs.

	Analytes	Retention time (min)
1	AM-2233	3.82
2	AM-1220	4.10
3	5F-ABICA	4.73
4	5F-MPP-PICA	6.23
5	NM-2201	6.56
6	5F-PB-22	6.58
7	MDMB-4en-PINACA	7.34
8	JHW-250	7.46
9	JHW-073	7.56
10	JHW-018	7.96
11	JHW-081	8.10
12	JHW-210	8.60
13	JHW-370	8.61

Table S13. Monitoring parameters of UPLC-MS/MS.

Analytes	Q_1	Q_3	DP (volts)	EP (volts)	CE (volts)	CXP (volts)
5F-PB-22	377.2	232.1	120	10	15	25
	377.2	144	120	10	54	25
5F-ABICA	348.2	232.2	30	10	20	10
	348.2	331.2	30	10	14	10
MDMB-4en-PINACA	358.2	213	120	10	24	10
	358.2	298.2	120	10	21	10
NM-2201	232.2	144.1	100	10	30	10
	232.2	116	100	10	47	10
JHW-073	328.2	155.1	162	10	30	10
	328.2	127	162	10	62	10
JHW-250	336.2	121.2	170	10	25	10
	336.2	200.1	170	10	32	10
AM-2233	459.1	98.2	196	10	33	10
	459.1	112.1	196	10	27	10
AM-1220	383.2	112.2	195	10	30	10
	383.2	127.1	195	10	77	10
JHW-018	342.2	155.2	160	10	31	10
	342.2	127.1	160	10	62	10
JHW-370	382.2	155.2	160	10	25	10
	382.2	127.1	160	10	75	10
JHW-210	370.2	183.1	181	10	40	10
	370.2	214.1	181	10	35	10
JHW-081	372.2	185.1	199	10	32	10
	372.2	214.1	199	10	35	10
5F-MPP-PICA	411.2	232.1	104	10	17	10
	411.2	144	104	10	57	10

References

[1] A. K. Rappé, C. J. Casewit, K. S. Colwell, W. A. Goddard, W. M. Skiff, *J. Am. Chem. Soc.* **1992**, *114*, 10024.

[2] S. Nosé, *J. Chem. Phys.* **1984**, *81*, 511.

[3] J. P. Perdew, K. Burke, M. Ernzerhof, *Phys. Rev. Lett.* **1996**, *77*, 3865.

[4] T. Lu, F. Chen, *J. Comput. Chem.* 2012, 33, 580.

[5] H. Martínez-Pérez-Cejuela, M. Conejero, P. Amorós, J. E. Haskouri, E. F. Simó-Alfonso, J. M. Herrero-Martínez, S. Armenta, *Anal. Chim. Acta*, **2023**, *1246*, 340887.

[6] X. Fan, J. Zhang, X. Fu, B. Zhou, Z. Xu, H. Huang, S. Han, X. Li, *Sci. Total Environ.* **2022**, *827*, 154267.

[7] C. E. O'Rourke, B. Subedi, *Environ. Sci. Technol*. **2020**, *54*, 6661.

[8] R. Bade, G. Eaglesham, K. M. Shimko, J. Mueller, *Talanta*, **2023**, *251*, 123767.

[9] Y. Shi, M. Liu, X. Li, N. Xu, S. Yuan, Z. Yu, P. Xiang, H. Wu, *J. Chromatogr. A*, **2022**, *1663*, 462743.

[10] P. Liu, W. Liu, H. Qiao, S. Jiang, Y. Wang, J. Chen, M. Su, B. Di, *Anal. Chim. Acta*, **2022**, *1226*, 340170.

[11] A. J. Pandopulos, B. S. Simpson, R. Bade, J. W. O'Brien, M. K. Yadav, J. M. White, C. Gerber, *Environ. Sci. Pollu.t Res.* **2021**, *28*, 59652.

[12] A. Cannaert, M. M. R. Fernández, E. L. Theunissen, J. G. Ramaekers, S. M. R. Wille, and C. P. Stove, *Anal. Chem.* **2020**, *92*, 6065.

[13] A. J. Pandopulos, R. Bade, J. W. O'Brien, B. J. Tscharke, J. F. Mueller, K. Thomas, J. M. White, C. Gerber, *Talanta,* **2020**, *217*, 121034.

[14] P. Tomai, A. Gentili, R. Curini, R. Gottardo, F. Tagliaro, S. Fanali, *J. Pharm. Anal.* **2021**, *11*, 292.

[15] C. Deriu, I. Conticello, A. M. Mebel, B. McCord, *Anal. Chem.* **2019**, *91*, 4780.

[16] J. Sánchez-González, S. Odoardi, A. M. Bermejo, P. Bermejo-Barrera, F. S. Romolo, A. Moreda-Piñeiro, S. Strano-Rossi, *J. Chromatogr. A*, **2018**, *1550,* 8.

[17] M. Pan, P. Xiang, Z. Yu, Y. Zhao, H. Yan, *J. Chromatogr. A*, **2019**, *1587*, 209.

[18] C. Montesano, G. Vannutelli, V. Piccirilli, M. Sergi, D. Compagnone, R. Curini, *Talanta*, **2017**, *167*, 260.

[19] T. Mostowtt, B. McCord, *Talanta*, **2017**, *164*, 396.
